# Integrated Metabolomic and Proteomic Analyses Reveal Altered Iron Homeostasis and Immunometabolic Remodeling in the Spleen of *Solea senegalensis* during Vibriosis

**DOI:** 10.1021/acs.jafc.6c02961

**Published:** 2026-10-19

**Authors:** Larissa Buedenbender, Miguel Balado, Diego Rey-Varela, Marta A. Lages, Patricia Fernández Puente, Jaime Rodríguez, Manuel L. Lemos, Anthony R. Carroll, Carlos Jiménez

**Affiliations:** † Departmento de Química, Facultad de Ciencias and CICA - Centro Interdisciplinar de Química e Bioloxía, 16737 Universidade da Coruña, A Coruña 15071, Spain; ‡ Department of Microbiology and Parasitology, Aquatic One Health Research Center (ARCUS), 16780 University of Santiago de Compostela, Santiago de Compostela 15782, Spain; § Proteomics Laboratory, CICA - Centro Interdisciplinar de Química e Bioloxía, Universidade da Coruña, A Coruña 15071, Spain; ∥ School of Environment and Science, Griffith University, Southport, Queensland 4222, Australia; ⊥ Department of Aquaculture and Blue Biotechnology, Centro Oceanográfico de Vigo (COV), Instituto Español de Oceanografía - Consejo Superior de Investigaciones Científicas (IEO−CSIC), Vigo 36390, Spain

**Keywords:** vibriosis, *Solea senegalensis*, aquaculture, infection, multiomics, biomarker discovery

## Abstract

*Vibrio anguillarum* is a major pathogen impacting *Solea senegalensis* aquaculture. While host–pathogen competition for iron drives disease progression, the *in vivo* molecular evidence linking iron homeostasis and immune responses in fish remains limited. Here, we integrated metabolomic and proteomic analyses to characterize the splenic response of *S. senegalensis* during experimental vibriosis. Multiomics integration revealed disruption of iron homeostasis alongside coordinated immunometabolic reprogramming. We detected the bacterial siderophore piscibactin directly in spleen tissue of sole exhibiting advanced stages of vibriosis, providing *in vivo* evidence of siderophore-mediated iron acquisition. Infection-driven metabolic shifts in lysine degradation, nucleotide metabolism, and mitochondrial energy pathways were correlated with heightened cellular proliferation and metabolic stress. Integrative analysis identified metabolites including 2-aminoadipic acid, saccharopine, and methylsuccinic acid, together with iron-regulatory proteins (e.g., STEAP4) as candidate biomarkers. These findings enhance our understanding of vibriosis-associated immunometabolic responses and provide a systems-level framework for future disease monitoring and management in aquaculture.

## Graphical abstract

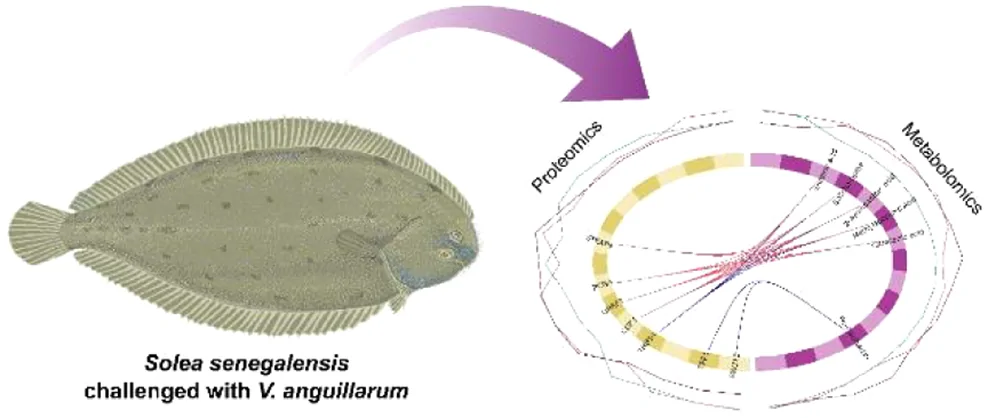

## 1 Introduction

The increasing global demand for food, together with declining wild fish stocks driven by overfishing and environmental degradation, has positioned aquaculture as one of the fastest-growing sectors of food production. However, infectious disease outbreaks remain a major challenge to aquaculture sustainability, resulting in significant economic losses.^1^ Although disease management largely relies on the implementation of vaccination programs, vaccines often require frequent reformulation and remain unavailable or ineffective for many host–pathogen systems.^2^ This persistent vulnerability highlights the need for improved approaches for disease prevention, early detection, and the identification of novel therapeutic targets.

The Gram-negative bacterium *Vibrio anguillarum* is the main cause of vibriosis in aquaculture and affects a wide range of marine species, including *Solea senegalensis* (Senegalese sole), an economically important species in Mediterranean and Atlantic fish farms.^3^ Infection typically occurs via ingestion or mucosal invasion, followed by systemic dissemination that often results in hemorrhagic septicemia and high mortality rates.^4^ Although vaccines against vibriosis are available, their efficacy varies across strains, and the emergence of vaccine-resistant strains of *V. anguillarum* represents an ongoing threat.^5,6^ Conventional diagnostics rely on clinical inspection and culture-based methods, which are laborious, time-consuming, and poorly suited for early infection.^7^ Molecular-level biomarkers could therefore improve disease monitoring and management.

Advances in high-throughput omics technologies have transformed the investigation of host–pathogen interactions by enabling systems-level analysis.^8^ Integrated proteomic and metabolomic analyses are particularly informative, as they capture dynamic biochemical and regulatory processes that are not always inferred from transcriptomic data alone.^9,10^ Such multiomics approaches facilitate biomarker discovery, elucidation of disease mechanisms, and identification of potential therapeutic targets.^8^ Despite these advantages, multiomics studies in aquaculture remain limited, particularly for early stage bacterial infections where rapid physiological reprogramming may dictate disease outcome.

Iron homeostasis plays a central role in host–pathogen interactions and shapes infection outcome as iron is essential for both host and pathogen metabolism. During infection, vertebrate hosts restrict iron availability through nutritional immunity, sequestering iron via proteins such as transferrin, ferritin, and lactoferrin.^11,12^ In response, pathogens have evolved high-affinity iron acquisition strategies, including siderophore-mediated uptake systems and specialized iron transport mechanisms, to scavenge iron from host sources and overcome iron limitation.^13,14^ In teleost fish, the spleen is a major immune organ involved in hematopoiesis, antigen presentation, phagocytosis, and erythrophagocytosis.^15^ It also serves as a key site for iron recycling, where macrophages sequester and store iron to limit pathogen access, while splenic cytokine signaling coordinates the inflammatory response.^16^ Despite the recognized importance of iron competition in vibriosis, the molecular mechanisms by which fish coordinate iron homeostasis, immune modulation, and metabolic adaptation during early *V. anguillarum* infection remain poorly characterized.

A growing body of work in mammalian and fish models demonstrates that infection triggers coordinated, organ-specific remodeling of metabolic and proteomics networks, reflecting the integrated nature of immune and metabolic responses.^17–21^ System-level immunometabolism studies further indicate that such reprogramming can profoundly influence disease trajectory, tolerance, and pathogen persistence.^22^ However, integrated analyses of metabolic and proteomic responses in teleost spleen during bacterial infection remain scarce. Given the spleen’s critical role as a secondary lymphoid organ and its involvement in iron handling, resolving its molecular response during infection is essential for understanding host–pathogen interactions in fish.

In this study, we applied an integrated metabolomic and proteomic approach to characterize the splenic response of *S. senegalensis* to experimental *V. anguillarum* infection, with a focus on iron homeostasis and immune regulation. By profiling infection-associated changes in metabolites and proteins, this multiomics study provides holistic insights into host–pathogen interactions during vibriosis and identifies candidate biomarkers for improved disease diagnosis and management in aquaculture.

## 2 Materials and Methods

### 2.1 Infection Challenge

The experimental infection challenge involved three groups of *S. senegalensis* fingerlings (n = 25 per group, ∼15 g each), randomly assigned and maintained in 100 L tanks with seawater recirculation and aeration. All fish were acclimated for 2 weeks and fed daily. The high-dose and low-dose groups received intraperitoneal injections of 100 μL PBS containing *V. anguillarum* RV22 at 5 × 10^5^ and 5 × 10^4^ CFU/fish, respectively, following a previously described protocol.^23^ The control group received PBS without bacteria. Fish were monitored daily for 7 days for signs of vibriosis and mortality. Dead fish were promptly removed. On day four, six fish per group were euthanized, and spleen samples were collected. Infection was confirmed by plating spleen samples on Tryptic-Casein Soy Agar with 1% NaCl (TSA-1), incubating at 25 °C for 24 h, and performing biochemical characterization of the recovered bacteria. The experiment was conducted at the Experimental Aquarium of the University of Santiago de Compostela in accordance with EU Directive 2010/63/EU on animal welfare. The protocol was approved by the Bioethics Committee of the University of Santiago de Compostela (ref. 15004/2022/003).

### 2.2 Sampling

Spleen tissues were snap frozen in liquid nitrogen and stored at −80 °C. To ensure consistency, samples were lyophilized and homogenized with a spatula. For proteomic analysis, 1–2 mg of lyophilized tissue were weighed and precise weights were recorded. For metabolomics, the remaining tissue (10–110 mg) was extracted with organic solvent.

### 2.3 Metabolomic Analysis

#### 2.3.1 Sample Preparation

The lyophilized tissue samples were extracted with cold MeOH:H_2_O (4:1) containing 100 μM FeCl_3_·6H_2_O + internal standard (100 μM 2-chloro-l-phenylalanine). One milliliter was added to each sample as well as six extraction blanks, that were then vortexed for 2 min, sonicated in an ice-bath for 10 min and incubated for 1 h at −20 °C. The samples were centrifuged at 20,000*g* for 10 min at 4 °C and supernatants were collected. The extraction procedure was repeated twice with 1 mL cold MeOH:H_2_O (4:1). For each sample, all supernatants were combined and 1 mL of ACN was added for protein precipitation overnight. The following morning, the samples were spun down at 20,000*g* for 10 min at 4 °C. Supernatants were collected in preweighed amber vials, dried under an N_2_ stream, and stored at −20 °C until analysis.

#### 2.3.2 LC-MS/MS Analysis

Untargeted metabolomics was performed on an Xevo G3 QTOF mass spectrometer system coupled to an ACQUITY UPLC system (Waters, Milford, MA, USA). Chromatographic separation was achieved by injecting (10 μL) the sample onto an ACQUITY UPLC HSS T3 column (1.8 μm, 2.1 × 100 mm). The mobile phase consisted of H_2_O containing 0.1% formic acid (A) and acetonitrile containing 0.1% formic acid (B). The gradient elution procedure was as follows: 0.0–0.5 min, 1% B; 0.5–10.0 min, 1–100% B; 10.0–12.0 min, 100% B; 12.0–12.5 min, 100–1% B; 12.5–15.0 min, 1% B. The column temperature was 40 °C and the flow rate was set to 0.5 mL/min. Mass spectra were obtained in both positive and negative ionization modes with scan time 1 s and scan mass range 80–1200 Da. The capillary voltages were 3.0 kV (positive mode) and 2.5 kV (negative mode), and the sample cone voltage was 40 V. The desolvation temperature and ion source temperature were 500 and 100 °C, respectively. Nitrogen was used as the carrier gas with a 1000 L/h flow rate for desolvation gas and a 50 L/h flow rate for sample cone gas. Data were recorded in continuum mode using MS^E^. The low collision energy was 6 eV, and the high collision energy was changed repeatedly from 20 to 50 eV. Leucine-enkephalin was used as LockMass solution during data collection. The UPLC-QTOF-MS system was controlled by MassLynx 4.2 software (Waters, Milford, MA, USA).

#### 2.3.3 Metabolomics Data Analysis

The raw mass spectrum data were converted to abf format by Reifycs ABF converter software (https://www.reifycs.com/AbfConverter/) and imported into MS-DIAL software (version 4.9) for peak alignment, peak picking, normalization, deconvolution, and compound identification.^24^ The main parameter settings are detailed in Table S1. Metabolites were identified against the MoNA-LC-MS/MS (https://mona.fiehnlab.ucdavis.edu/), HMDB,^25^ GNPS^26^ databases. Additionally, data were exported in msp format and annotated using SIRIUS.^27^

For statistical analysis, samples were quantile normalized, log-transformed and Pareto scaled using MetaboAnalyst 6.0.^28^ Principal component analysis (PCA) was performed to gain an overview of the relationships among the metabolomics profiles of the different treatments. A sparse partial least-squares-discriminant analysis (sPLS-DA) was used as an exploratory supervised dimensionality-reduction approach to visualize group discrimination. Model tuning was performed by evaluating different numbers of latent components and variable selection sizes per component. A 5-fold cross-validation strategy was used to assess model stability and sample discrimination while accounting for the limited cohort size and reducing variability associated with fold partitioning. Two-component models were selected as they provided a balance between classification performance and model simplicity. The number of variables retained per component was evaluated in the range of 5–10 variables; however, a more parsimonious configuration (5 variables per component) was selected as the final model to reduce model complexity and minimize the risk of overfitting while maintaining comparable cross-validation performance. Differential metabolite analysis was performed using a Student’s *t*-test followed by Benjamini–Hochberg false discovery rate (FDR) correction. Metabolites were considered significantly differentially expressed when meeting both an adjusted *p*-value threshold (FDR-adjusted *p* <0.05) and an absolute fold-change threshold (FC ≥2.0). Results were visualized in volcano plots. Significant differentially expressed metabolites (DEM) were used for pathway enrichment analysis. Because no dedicated Kyoto Encyclopedia of Genes and Genomes (KEGG) annotation resource for *S. senegalensis* is currently available, *Danio rerio* was used as the closest well-annotated teleost reference.^29^ Consequently, all pathway assignments should be regarded as putative.

### 2.4 Proteomics Analysis

#### 2.4.1 Sample Preparation

Protein extractions were performed from 1 mg of lyophilized sample. Samples were homogenized with 100 μL lysis buffer (2% SDS/6 M urea/25 mM ammonium bicarbonate) and vortexed until a homogeneous solution was obtained and then sonicated for 3 min. Centrifugation at 13,000 rpm was applied to remove cell debris and other insoluble materials. Proteins were then precipitated overnight with acetone at −20 °C and further resuspended with 6 M urea/2 M thiourea/25 mM ammonium bicarbonate. Protein concentration was estimated through a Bradford assay in a spectrophotometer at 590 nm upon calibration with a BSA standard curve. The quality of the samples was confirmed using SDS-PAGE gel and silver nitrate staining.

#### 2.4.2 LC-MS/MS Analysis

The protein samples were subjected to in-solution digestion prior to LC-MS/MS analysis. First, equal amounts of each sample (approximately 10 μg) were reduced with 10 mM dithiothreitol for 1 h at 37 °C and subsequently alkylated with 50 mM iodoacetamide for 45 min at room temperature in the dark. The samples were digested with sequencing grade modified trypsin (Promega) at a 1:40 enzyme-to-substrate ratio. After 16 h of digestion at 37 °C, the samples were acidified with 10% trifluoroacetic acid. The digested peptides were desalted using in-house prepared stage tips (3 M Empore SPE-C18 disk, 47 mm, Sigma-Aldrich) and finally dried under speed-vacuum. Dried eluates were reconstituted in water with 2% ACN and 0.1% formic acid for direct nLC-MS. The peptide mixture (200 ng) was analyzed on a nanoElute nanoflow LC (Bruker Daltonics) coupled to a high-resolution TIMS-QTOF (timsTOF Pro, Bruker Daltonics) with a CaptiveSpray ion source. Liquid chromatography was performed at 50 °C and with a constant flow of 400 nL/min on a reversed-phase PepSep column (15 cm × 75 μm) with a pulled emitter tip, packed with 1.9 μm C18-coated porous silica beads. Chromatographic separation was carried out using a linear gradient of 5–35% buffer B (100% ACN and 0.1% FA) over 40 min. After ESI ionization, peptides were analyzed in data-independent mode (DIA) with the Parallel Accumulation–Serial Fragmentation (PASEF) enabled. The isolation width was set as 26 *m*/*z* and precursor ions of 400–1,200 *m*/*z* and ion mobility of 0.60–1.60 V s/cm^2^ were monitored with 32 windows. The collision energy was programmed as a function of ion mobility, following a straight line from 20 eV for 1/K_0_ of 0.6 V s/cm^2^ to 59 eV for 1/K_0_ of 1.6 V s/cm^2^.

#### 2.4.3 Proteomics Data Analysis

Protein identification and label free quantification (LFQ) were performed using DIA-NN (version 1.8.1).^30^ A library-free search strategy was applied using the UniProtKB *Solea senegalensis* proteome. Due to the low number of reviewed protein entries available for *S. senegalensis* (7 validated SwissProt and 4501 unreviewed TrEMBL entries), the analysis was complemented with the well-annotated *Danio rerio*proteome (3352 SwissProt and 45920 TrEMBL entries; downloaded 07–03–2024 from UniProtKB) to facilitate homology-based protein annotation and functional interpretation. Minimum peptide length was set to seven amino acids. *N*-terminal methionine excision and cysteine carbamidomethylation were specified as fixed modifications, while variable methionine oxidation was enabled with a maximum of three variable modifications per peptide. Trypsin/P was selected as digestion enzyme, and identifications were filtered using a 1% false discovery rate (FDR) threshold at the peptide level. For exploratory statistical analysis, protein group abundance data were log-transformed and autoscaled prior to PCA, sPLS-DA, and volcano plot analyses. Multivariate analyses and model validation were conducted using the same cross-validation and parameter selection strategy as described for the metabolomics data set. Differentially expressed proteins (DEPs) were identified based on fold change thresholds adapted to the downstream analysis requirements (FC ≥2.0, for stringent differential analysis and FC ≥1.5 for functional analysis), together with statistical significance (adjusted *p*-value <0.05). Functional enrichment analysis was performed using the R package clusterProfiler.^31^ Following completion of the original data analysis, proteins discussed in the Results and Discussion sections were retrospectively verified against entries in the recently released *S. senegalensis* proteome derived from genome sequencing (UniProt UP000693946; 44389 unreviewed TrEMBL entries; downloaded 20–09–2026 from UniProtKB), based on protein descriptions and homology (Supplementary Table S3B–C).

### 2.5 Multiomics Integration for Biomarker Discovery

Metabolomic and proteomic data sets were integrated using the Data Integration Analysis for Biomarker discovery using Latent cOmponents (DIABLO) framework implemented in the R package mixOmics.^32^ To focus the integration on biologically relevant immunometabolic signatures, only differentially expressed metabolites (DEMs) and proteins (DEPs) meeting statistical significance (adjusted *p* <0.05) and fold-change thresholds (FC ≥2.0) were included. This feature-selection strategy was intended to reduce dimensionality and improve biological interpretability. The DIABLO analysis should be considered exploratory rather than an independent validation of group discrimination.

Technical replicates from the metabolomics data set were merged using the “Merging Technical Replicates” module in MetaboAnalyst 6.0 by simple arithmetic mean. The DEM and DEP data sets were log-transformed and autoscaled to account for the dynamic ranges of different omics platforms. A design matrix was constructed based on a preliminary sPLS correlation analysis (*r* = 0.847), facilitating a supervised integration of metabolomic and proteomic blocks.

Model tuning was performed using 6-fold cross-validation with 50 repetitions. The optimal number of latent components was determined by minimizing the Balanced Error Rate (BER), resulting in a three-component model. To reduce model complexity and limit overfitting, feature selection was constrained by a parsimony criterion, retaining five variables per data block per component. Model significance was assessed using permutation testing (200 permutations), in which class labels were randomly shuffled to generate a null distribution of BER values. The observed model performance was significantly better than expected by chance (*p* < 0.05). Features contributing to the selected latent components were further investigated as candidate infection-associated biomarkers, and strong cross-omics associations (|*r*| > 0.85) were visualized using a Circos plot.

The diagnostic potential of the candidate metabolite and protein biomarkers was further assessed via Receiver Operating Characteristic (ROC) curve analysis using the R package pROC.^33^ ROC curves for each individual candidate biomarker were constructed by utilizing the relative metabolite and protein abundances. The effects of the candidate biomarkers were evaluated based on the area under the curve (AUC) (0.9–1.0 = excellent; 0.8–0.9 = good; 0.7–0.8 = fair; 0.6–0.7 = poor; and 0.5–0.6 = fail). The ROC analyses were performed on the same cohort used for feature discovery and should therefore be interpreted as exploratory.

## 3 Results

### 3.1 *V. anguillarum* Infection and Disease Characterization in *Solea senegalensis*

To characterize the metabolic response of *S. senegalensis* to *V. anguillarum*, an infection challenge was conducted using two bacterial doses. The low-dose group received 5 × 10^4^ CFU/fish, corresponding to a previously estimated lethal dose 50% (LD_50_),^23^ while the high-dose group (5 × 10^5^ CFU/fish) represented a 10-fold increase intended to induce a more severe and accelerated infection phenotype. At 4 days postinfection, 40% mortality was recorded in the high-dose group, along with visible signs of disease, including hemorrhages in the fins and petechiae (Figure 1A,B). In contrast, the low-dose group showed delayed disease progression, with clinical symptoms appearing later and cumulative mortality reaching approximately 20% by day seven (Figure 1A). For metabolomic and proteomic profiling, spleen samples from *S. senegalensis* were collected 4 days postinfection. At this point, the high-dose group exhibited greater infection severity than the low-dose group. *V. anguillarum* RV22 was consistently recovered from all sampled fish in both infected groups, with higher bacterial loads observed in organs from the high-dose group, further supporting differences in infection severity (Figure S1). No clinical signs of vibriosis, mortality, or bacterial recovery were observed in the control group (PBS injected). Thus, the high-dose/low-dose comparison was used as a proxy for infection severity rather than temporal disease progression.

**Figure 1:**
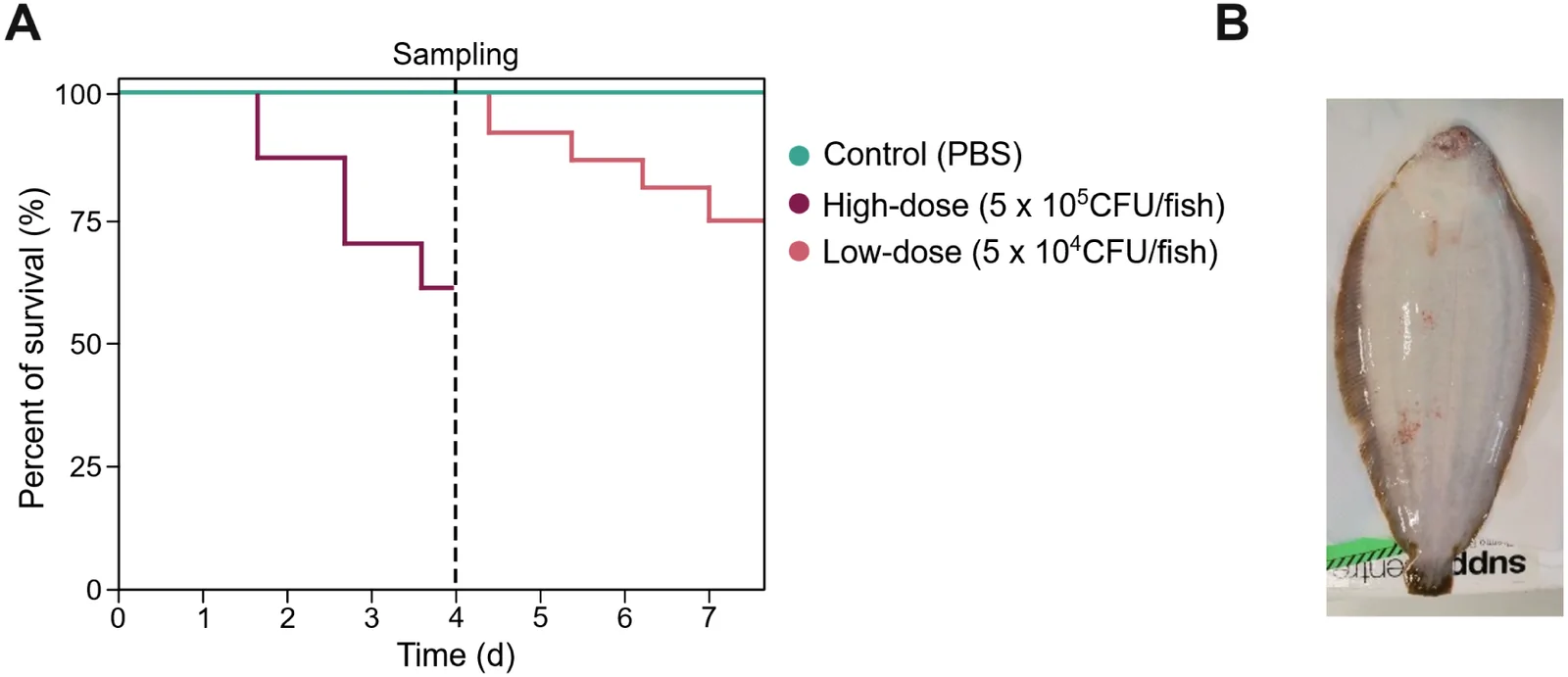
Infection challenge (A) Kaplan–Meier survival curves of *S. senegalensis* infected with *V. anguillarum* at low (5 × 10^4^ CFU/fish) and high (5 × 10^5^ CFU/fish) doses. The dashed line marks the sampling point (day 4), when six live fish per group were sampled to collect spleen tissues. (B) *S. senegalensis* showing external signs of vibriosis.

### 3.2 Metabolome Profile of *V. anguillarum*-Infected *S. senegalensis* Spleen Tissues

LC-MS/MS-based metabolomics data of *S. senegalensis* spleen extracts revealed differences between the control and infected fish. Principal component analysis (PCA) indicated some separation between the groups with PC1 accounting for 22.8% and 29.8% of the total variance in the positive and negative ionization mode metabolomic data sets, respectively (Figure 2A), confirming that the primary variance is driven by infection severity rather than baseline individual heterogeneity. The optimized sPLS-DA model (2 components, 5 variables/component) further confirmed discriminatory performance between experimental groups based on 5-fold cross-validation (Figure 2B). For the positive and negative ionization mode data sets, the classification error rates were 19.7% and 18.2% for the first component and 4.2% and 4.3% for the second component, respectively, indicating that a subset of selected features contributed strongly to group separation (Figures 2B and S2). In the low-dose group, 741 differentially expressed metabolites (DEMs) were detected (346 upregulated and 395 downregulated) in positive ionization mode and 675 DEMs (327 up, 348 down) in negative ionization mode (Figure S3). In the high-dose group, 1043 DEMs (509 up, 534 down) were identified in positive ionization mode and 915 (457 up, 458 down) in negative ionization mode (Figure S3). DEMs were annotated using multiple MS^2^ databases which resulted in putative identification of 223 significantly altered metabolites (Figure 2C–D, Table S2). These metabolites predominantly belonged to amino acids, peptides and analogues, nucleotides, and different lipid classes (Figure 2C,D and S4). Notably, piscibactin, one of the siderophores produced by *V. anguillarum* (Figure 2E),^34^ was detected in the high-dose group compared with control (log_2_(FC) = 4.4544, adjusted *p-*value = 1.67E^–6^), providing direct metabolomic evidence of siderophore presence in host tissue during infection.

**Figure 2:**
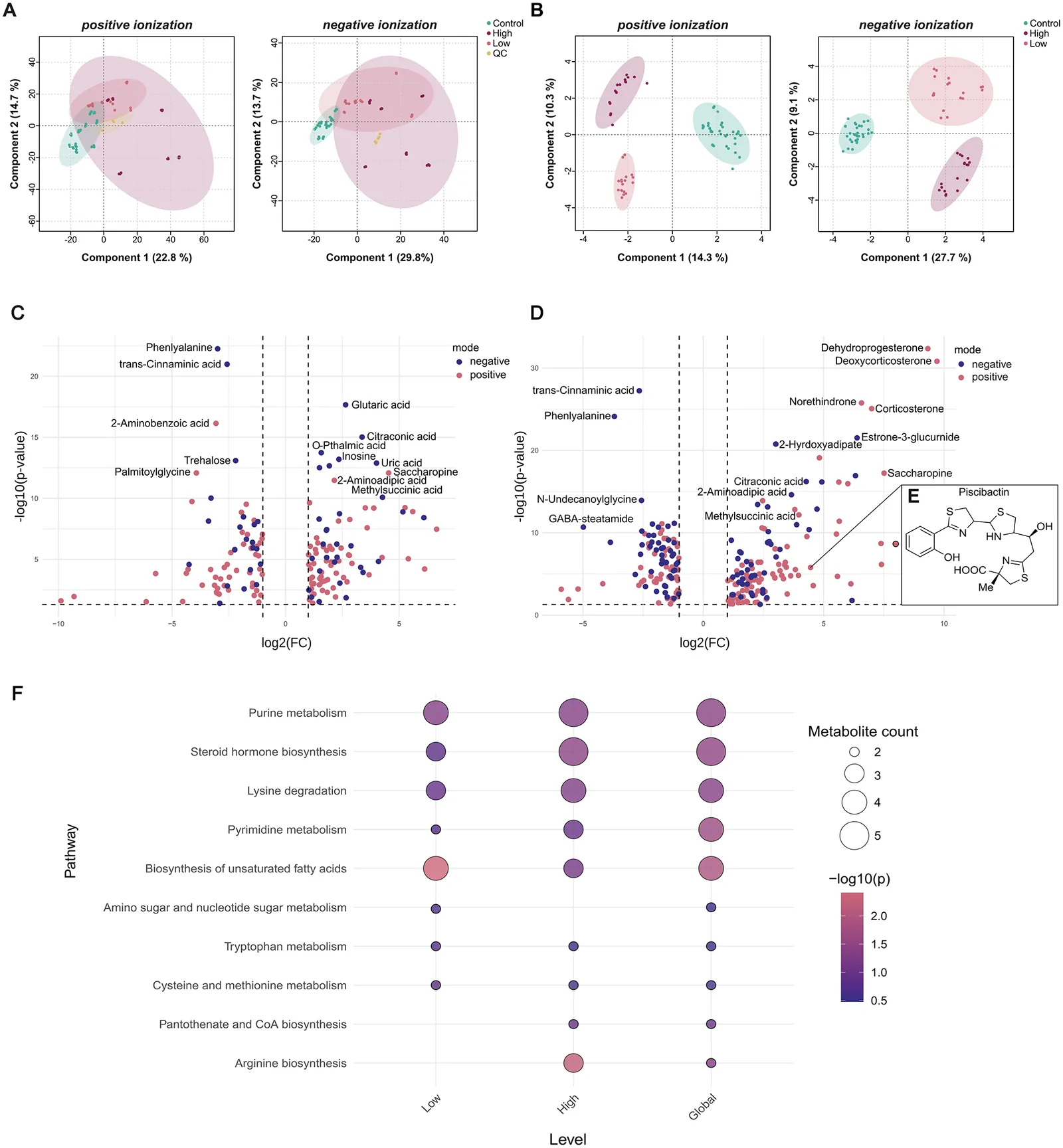
Untargeted metabolomics analyses in the spleen of *S. senegalensis* during vibriosis, showing separation between control, low- and high-dose groups. (A) PCA score plot of positive and negative ionization mode data, both showing group separation with pooled QC samples well centered in the plot; colored ellipses represent the 95% confidence intervals. (B) sPLS-DA score plot of positive and negative ionization mode data showing clear group separation. (C) Volcano plot of the annotated differentially expressed metabolites (DEMs; FC ≥2.0, adjusted *p* < 0.05) in the low-dose group showing 87 annotated metabolites that were upregulated and 72 downregulated. (D) Volcano plot of the annotated DEMs in the high-dose group, with 131 upregulated and 93 downregulated metabolites. (E) Chemical structure of the *V. anguillarum* siderophore piscibactin.^34^ (F) Functional analysis of differentially expressed metabolites (DEMs) in the spleen of *S. senegalensis* during vibriosis. KEGG pathway enrichment was based on metabolites with FC ≥2.0, *p*-value <0.05. The “Global” category includes all infected samples, combining low- and high-dose groups. Dot color represents statistical significance, and dot size the number of DEMs associated with each enriched pathway.

Pathway analysis of DEMs was performed against the KEGG zebrafish (*Danio rerio)* database to identify metabolic processes altered during infection. Several pathways were significantly enriched, including nucleotide metabolism (purine and pyrimidine metabolism), lipid metabolism (steroid hormone biosynthesis and biosynthesis of unsaturated fatty acids), and lysine degradation (Figure 2F). To a lesser extent, metabolites associated with other amino acid metabolism (tryptophan metabolism, cysteine and methionine metabolism, arginine biosynthesis), as well as amino sugar and nucleotide sugar metabolism and pantothenate and CoA biosynthesis were also detected (Figure 2F). Together, these results indicate dose-dependent metabolic reprogramming of the spleen during *V. anguillarum* infection.

### 3.3 *S. senegalensis* Spleen Proteome Profile During the Infection Process

Proteomic analysis of *S. senegalensis* spleen extracts initially resulted in the identification of 222 proteins using the UniProtKB *S. senegalensis* database, while the use of the well-annotated *D. rerio* proteome expanded protein identifications to 2294 proteins (Table S3A). Protein identifications derived from the zebrafish database were treated as putative orthologs and filtered using a stringent peptide-level false discovery rate (1% FDR) implemented in DIA-NN to minimize false-positive identifications. This cross-species mapping approach substantially expanded proteomic coverage and facilitated downstream functional interpretation. Retrospective comparison against the recently available *S. senegalensis* proteome (UniProt proteome UP000693946; downloaded 20–09–2026) showed that 77% and 80% of the DEPs identified in the low-dose and high-dose groups, respectively, could be linked to corresponding *S. senegalensis* proteome entries (Table S3B–C), providing additional support for the homology-based assignments and their functional interpretation. Importantly, key proteins discussed in this study, including STEAP4, PCNA, CDK1, and UHRF1, were among those successfully linked to corresponding *Solea* proteome entries.

Multivariate analysis of the proteomics data revealed clear separation between treatment groups, with both PCA and sPLS-DA distinguishing control samples from those of low- and high-dose infection groups (Figure 3A,B). Differential expression analysis identified 122 differentially expressed proteins (DEPs; FC ≥2.0, adjusted *p* < 0.05) between the low-dose infection group and controls, comprising 77 upregulated and 45 downregulated (Figure 3C). In the high-dose group, 208 DEPs were detected relative to controls, including 66 upregulated and 142 downregulated (Figure 3D).

**Figure 3:**
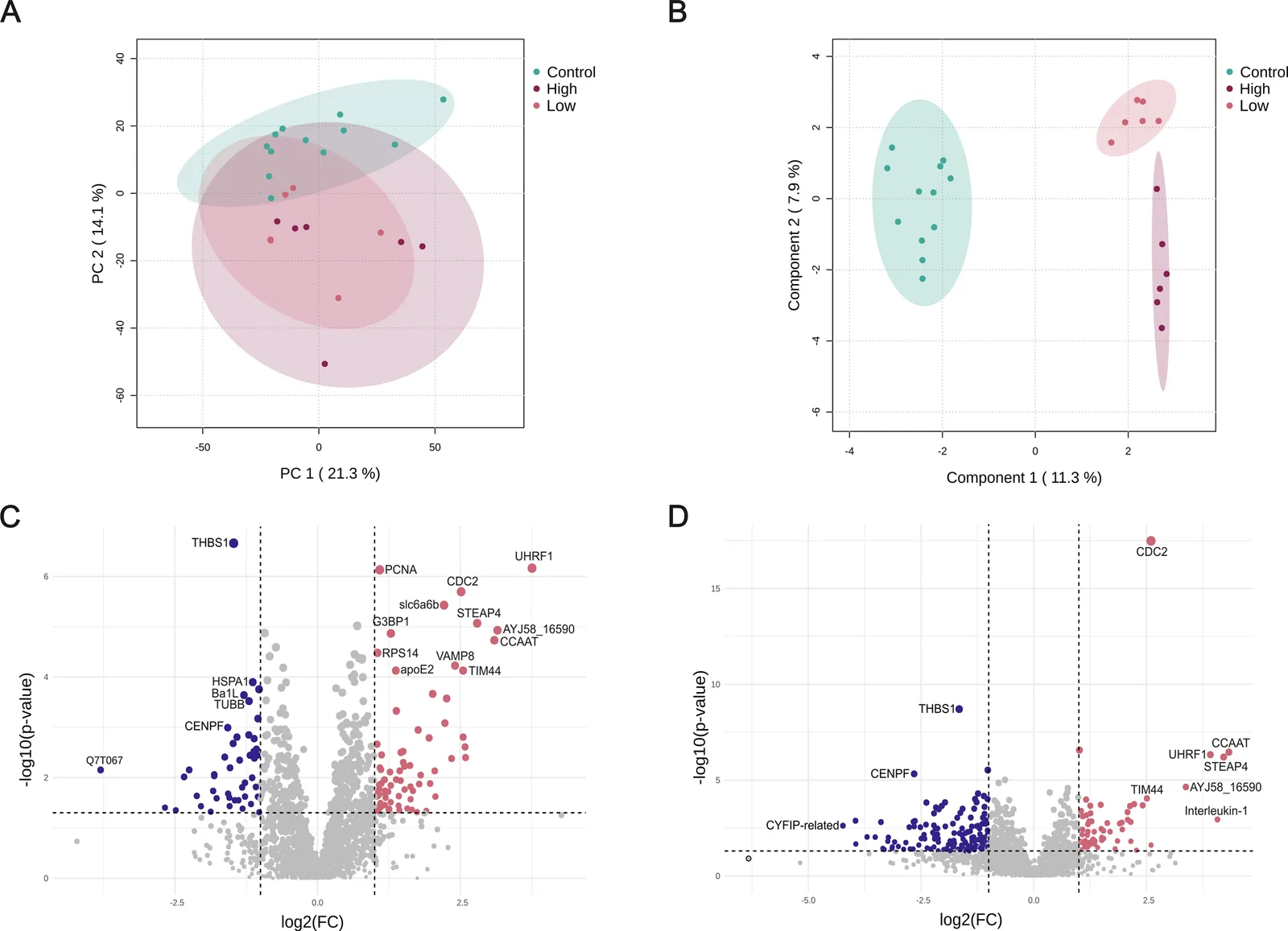
Shotgun proteomics analysis of spleen extracts from *S. senegalensis* infected with *V. anguillarum* and controls. (A) PCA scores plot and (B) sPLS-DA scores plot showing separation between the different treatment groups. Volcano plots showing differentially expressed proteins (DEPs; FC ≥2.0, adjusted *p* <0.05) between (C) the low-dose group and control, and (D) the high-dose group and control. Red dots represent upregulated proteins; blue dots represent downregulated proteins.

Functional analysis based on these putative ortholog assignments using Gene Ontology (GO) and KEGG databases revealed significant enrichment of biological processes associated with cellular repair, protein synthesis and energy metabolism (Figure 4A,B and S5–S7). Biological processes included DNA damage response, DNA-templated replication, and double-strand break repair, along with replication fork and minichromosome maintenance (MCM) complex, were highly enriched, indicating activation of DNA replication machinery. At the molecular function level, enrichment of DNA binding, helicase activity, nucleotide binding, and RNA polymerase activity further supported increased nucleic acid metabolism during infection. KEGG analysis across the different infection levels (low-dose, high-dose, and combined “global” groups) highlighted significant enrichment of carbon metabolism and ribosome, consistent with increased ribosome biogenesis and translational activity during *V. anguillarum* infection. Additional enrichment of oxidative phosphorylation, protein processing in the endoplasmic reticulum, and the citrate cycle (TCA cycle) further indicated heightened metabolic and biosynthetic activity in the spleen during infection.

**Figure 4:**
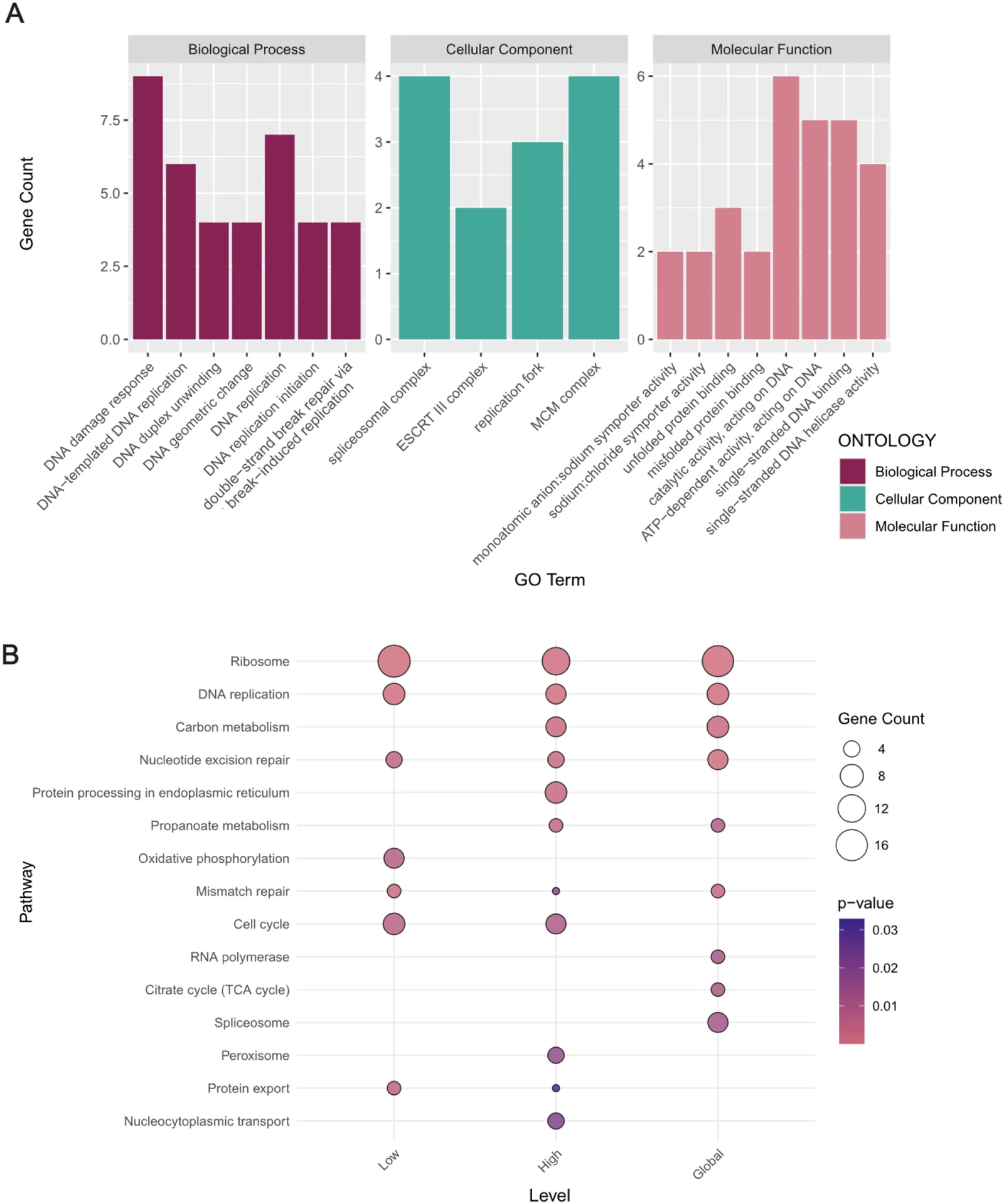
Functional analysis of differentially expressed proteins (DEPs) during vibriosis. (A) Enriched Gene Ontology (GO) terms for Molecular Functions, Cellular Components, and Biological Processes. (B) KEGG pathway enrichment analysis based on DEPs with FC ≥1.5 and raw *p* < 0.05 across different infection levels. The “Global” group includes all infected samples (low- and high-dose combined). Dot color indicates statistical significance (*p*-value); dot size corresponds to the number of proteins associated with each pathway. Note: Functional analyses are based on zebrafish annotations and are hence to be interpreted as putative orthologs.

### 3.4 Metabolome and Proteome Data Integration

To investigate the coordinated immunometabolic response of *S. senegalensis* to *V. anguillarum* infection, we integrated significant DEPs and DEMs (adjusted *p* <0.05, FC ≥2.0) using the Data Integration Analysis for Biomarker discovery using Latent cOmponents (DIABLO). This multivariate framework integrates omics data sets to identify the highly correlated variables that jointly discriminate between experimental groups.^32^ DIABLO analysis revealed a strong concordance between metabolomic and proteomic data sets, with the first latent component showing a high correlation between data blocks (*r* = 0.96) and clear separation of infected and control samples (Figures 5A and S8). Cross-validation indicated that the lowest error rate was achieved using three components (Figure S9), which were therefore retained for further analysis. For each component, the five most discriminative metabolites and proteins were selected based on model loadings (Figure S10). Correlations between selected features were visualized using a Circos plot with a correlation cutoff *r* = 0.85 (Figure 5B). This analysis identified 17 positive and six negative correlations involving six metabolites and seven proteins (Figures 5B,C and S11) as candidate biomarkers for vibriosis (Table 1).

**Figure 5:**
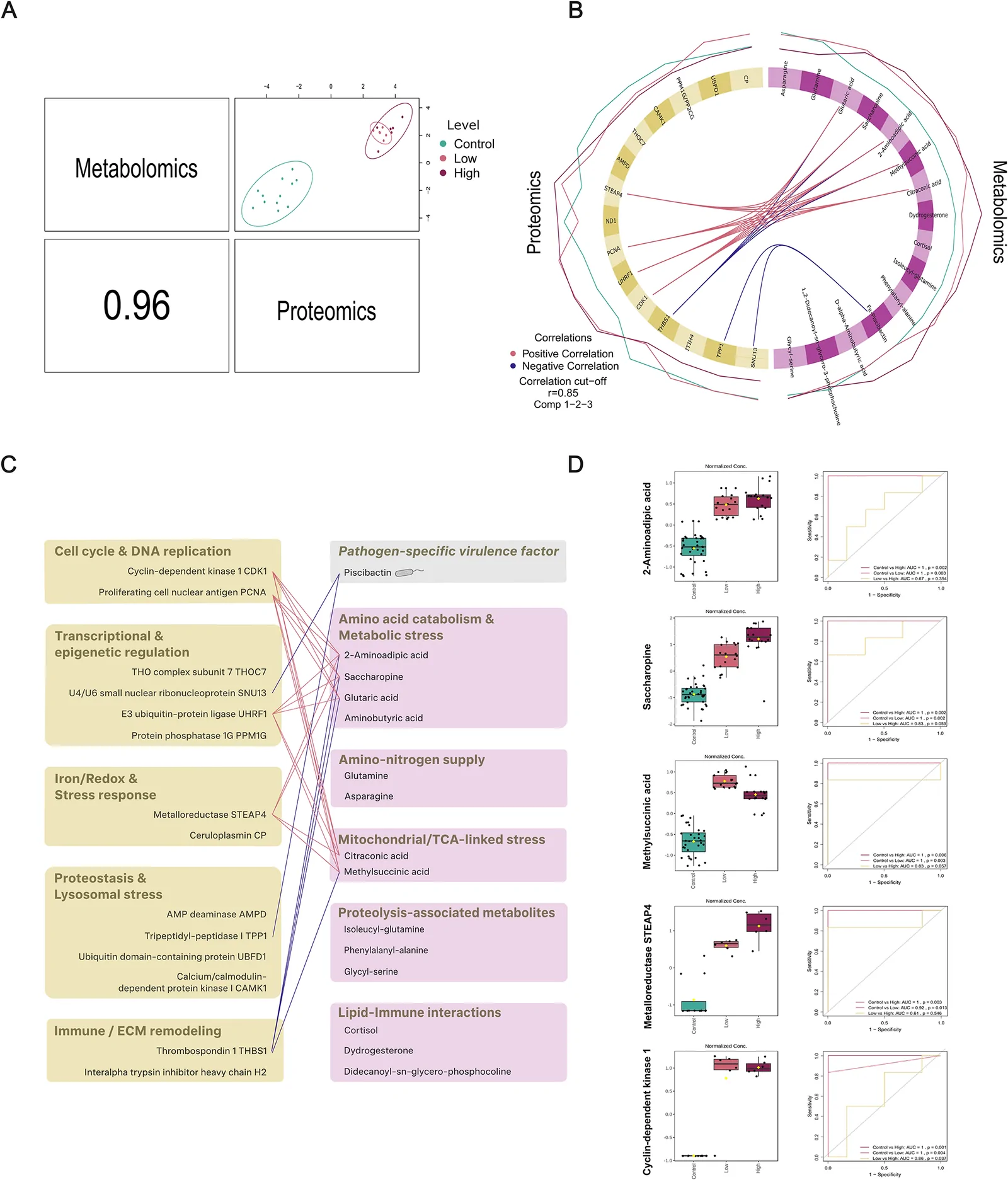
Integrated multiomics framework reveals coordinated molecular responses in *S. senegalensis* spleen during *V. anguillarum* infection. (A) Pearson correlation analysis between metabolomics and proteomics data sets across control, low-dose, and high-dose groups, illustrating the high cross-block covariance (*r* = 0.96). (B) Circos plot integrating proteomics and metabolomics analysis using DIABLO, showing strong cross-omics immunometabolic associations (|*r*| = 0.85) between the top-ranked discriminatory proteins and metabolites in the first three latent components. Internal connecting lines show positive (pink) or negative (blue) correlation between variables from different data blocks, while outer lines represent relative abundance across experimental groups. (C) Conceptual framework summarizing the functional categorization of the identified candidate biomarkers, mapping omics features to key biological pathways (e.g., iron homeostasis, cell cycle, and proteostasis). (D) Box plots showing normalized relative abundance and Receiver Operating Characteristic (ROC) analyses of selected candidate biomarker metabolites and proteins associated with *Vibrio* infection. Corresponding Areas under the Curve (AUC) values and adjusted *p*-values are presented. Note that ROC analyses should be interpreted as exploratory due to the absence of independent validation cohorts.

**Table 1:** Candidate Metabolite and Protein Biomarkers Identified Through Integrative Omics Analysis of Spleen Tissue from *S. senegalensis* Challenged with *V. anguillarum* ^a^

			**AUC (** *p*-value)
**Name**	**HMDB or UniProt ID**	**KEGG ID**	Control vs High/Control vs Low/Low vs High
**2-Aminoadipic acid**	HMDB0000510	C00956	**1.0 (0.002)/1.0 (0.003)/** **0.67 (0.354)**
**Saccharopine**	HMDB0000279	C00449	**1.0 (0.002)/1.0 (0.002)/** **0.83 (0.059)**
**Methylsuccinic acid**	HMDB0001844	n/a	**1.0 (0.006)/1.0 (0.003)/** **0.83 (0.057)**
Citraconic acid	HMDB0000634	C02226	1.0 (0.005)/1.0 (0.006)/0.64 (0.47)
Glutaric acid	HMDB0000661	C00489	0.68 (0.294)/0.87 (0.05)/0.76 (0.147)
Piscibactin	n/a	n/a	0.81 (0.1)/0.83 (0.05)/0.92 (0.014)
**Cyclin-dependent kinase 1 CDK1**	Q7T3L7	K02087	**1.0 (0.003)/0.92 (0.13)/** **0.61 (0.546)**
**Metalloreductase STEAP4**	F1Q9R5	K19876	**1.0 (0.001)/1.0 (0.004)/0.86 (0.037)**
Thrombospondin 1 THBS1	F1QL35	K16857	1.0 (0.003)/1.0 (0.003)/0.5 (0.894)
E3 ubiquitin-protein ligase UHRF1	E7EZF3	K10638	1.0 (0.002)/0.97 (0.005)/0.42 (0.999)
Proliferating cell nuclear antigen PCNA	Q9PTP1	K04802	1.0 (0.003)/1.0 (0.003)/0.5 (0.894)
Tripeptidyl-peptidase I TPP1	F8W2M8	K01279	0.89 (0.016)/0.47 (0.939)/0.86 (0.059)
U4/U6 small nuclear ribonucleoprotein SNU13	Q7ZV23	K12845	1.0 (0.001)/0.56 (0.78)/0.94 (0.01)

a HMDB/Uniprot and KEGG IDs are provided. Areas under the curve (AUC) from Receiver Operating Characteristic (ROC) analysis are presented. Candidate biomarkers with the highest discriminatory performance are presented in bold.

Many of the observed associations involved metabolites related to amino acid metabolism and immune- and cell cycle-related proteins. Intermediates in lysine degradation, such as glutaric acid, 2-aminoadipic acid, and saccharopine, showed strong positive correlations with proteins including cyclin-dependent kinase 1 (CDK1), proliferating cell nuclear antigen (PCNA), and the E3 ubiquitin-protein ligase UHRF1, proteins involved in cell cycle control and DNA methylation (Figure 5C). Similarly, the metalloreductase STEAP4, a protein involved in iron homeostasis and inflammatory responses, showed positive correlations with 2-aminoadipic acid, saccharopine, citraconic acid, and methylsuccinic acid (Figure 5C). In contrast, the pathogen-derived siderophore piscibactin exhibited negative correlations with tripeptidyl-peptidase I (TPP1) and U4/U6 small nuclear ribonucleoprotein (SNU13), indicating statistical associations with host proteins involved in lysosomal proteolysis and RNA splicing (Figure 5C).

### 3.5 Identifying Candidate Infection-Associated Biomarkers

To identify candidate biomarkers, Receiver Operating Characteristic (ROC) curve analyses were performed and the corresponding Areas Under the Curve (AUC) were calculated. Several metabolites (2-aminoadipic acid, saccharopine, citraconic acid, methylsuccinic acid) and proteins (STEAP4, THBS1, UHRF1, CDK1, PCNA) exhibited discriminatory potential (AUC > 0.9) to distinguish infected fish from controls (Figures 5D and S12–S13). Among these, 2-aminoadipic acid, saccharopine, and STEAP4 showed progressive increases with infection severity. ROC analysis comparing low- and high-dose infection groups further indicated high discriminatory performance for saccharopine and STEAP4 (Figure 5D). In contrast, methylsuccinic acid was specifically elevated in the low-dose infection group, supporting its prioritization as a candidate early stage biomarker for infection-associated immune activation (Figure 5D).

## 4 Discussion

Senegalese sole (*Solea senegalensis*) is an important aquaculture species; however, outbreaks of vibriosis caused by *Vibrio anguillarum* continue to compromise production despite increasing vaccination efforts. The molecular mechanisms underlying host responses to infection in this species remain poorly characterized. Through integrated metabolomic and proteomic analyses of spleen tissue, we identified coordinated molecular alterations associated with iron homeostasis, immunometabolic remodeling, and candidate biomarker signatures during *V. anguillarum* infection. Given the exploratory nature of this multiomics study, the sample size (n = 6 per group) was selected to balance biological replication with ethical considerations under the 3Rs principle and is comparable to similar recently published aquaculture omics studies.^35–40^

Our analyses revealed dose-dependent metabolic reprogramming in the spleen of *S. senegalensis* during *V. anguillarum* infection, with iron regulation emerging as a central feature of the host response.^41^ As samples were collected on day 4 postinfection, these molecular signatures represent a snapshot of the host response, and differences between infection groups may reflect both dose and infection severity rather than temporal disease progression. Increasing levels of iron-handling proteins, including STEAP4-like metalloreductase and ceruloplasmin, indicate active modulation of the iron redox balance during infection. STEAP4 facilitates intracellular iron utilization,^42^ while ceruloplasmin links metal homeostasis to inflammatory signaling.^43^ In parallel, pathogens deploy siderophore-mediated iron acquisition systems to overcome host-imposed iron limitation.^13,44^ Highly virulent *V. anguillarum* strains (e.g., RV22) produce both vanchrobactin and piscibactin, with piscibactin primarily contributing to virulence and vanchrobactin to environmental persistence.^23,45–47^ The detection of piscibactin and the recovery of viable *V. anguillarum* cells from infected spleen tissue provides direct *in vivo* evidence of siderophore presence during infection. Although this observation is consistent with previously proposed functional specialization among siderophores,^23^ differential systemic dissemination or accumulation cannot be excluded. Similar siderophore-associated signatures reported in other fish infections further underscore the importance of iron acquisition in bacterial pathogenesis.^48–51^ Together, host iron regulation and pathogen-derived siderophore signatures highlight the importance of iron competition during host–pathogen interactions.

Metabolomic profiling revealed immunometabolic remodeling, particularly in amino acid, nucleotide, lipid, and central carbon metabolism. Lysine degradation was prominent, with intermediates such as glutaric acid, 2-aminoadipic acid, and saccharopine showing association with infection. Lysine catabolism supports mitochondrial metabolism by supplying acetyl-CoA and reducing equivalents,^52,53^ while enrichment of purine and pyrimidine metabolism likely reflects increased nucleotide demand. Perturbations in lipid-related pathways further suggest membrane remodeling and metabolic adaptation under infection-associated stress. Collectively, these changes suggest coordinated immunometabolic activation. Metabolite-level pathway assignments are generally more conserved across teleosts than gene-level annotations, although species-specific differences cannot be excluded.

Proteomic analysis supported these findings, showing enrichment of DNA replication and repair, ribosome biogenesis, and protein synthesis pathways, consistent with increased cellular proliferation in the spleen. Although classical inflammatory pathways were not directly detected, these signatures align with immune activation.^54^ Due to limited annotation of the *S. senegalensis* proteome at the time of analysis, functional interpretation was complemented using the well-annotated *D. rerio* proteome.^55^ While this may introduce species-specific annotation bias,^56^ zebrafish-based functional assignments should be considered putative. Notably, retrospective comparison with the recently available *S. senegalensis* proteome linked 77–80% of the DEPs to corresponding *S. senegalensis* entries, including key proteins such as STEAP4, PCNA, CDK1, and UHRF1, further supporting the biological interpretation of the proteomic data.

Previous metabolomic and multiomics studies in fish challenged with *Vibrio* spp. and other pathogens have consistently identified alterations in amino acid metabolism, central carbon pathways, and redox balance as key features of the host response.^8,57–59^ In the present study, integration of proteomic and metabolomic data sets reinforced the coordinated nature of the splenic response and enabled prioritization of infection-associated molecular signatures. DIABLO analysis revealed significant associations between lysine degradation intermediates, including 2-aminoadipic acid and saccharopine, and cell cycle-related proteins such as CDK1, PCNA, and UHRF1, likely reflecting parallel responses to infection-induced stress rather than direct functional coupling. Notably, lysine degradation intermediates were also associated with STEAP4, linking amino acid catabolism with iron homeostasis and suggesting increased metabolic demands during immune activation.^60,61^ Although causality cannot be inferred, elevated levels of 2-aminoadipic acid have also been detected in flounders affected by edwardsiellosis,^62^ anemic catfish,^63^ and *Vibrio*-infected shellfish.^64^ Similarly, several studies have also observed elevated levels of STEAP4 in infected fish,^65–71^ supporting its role in infection-associated host responses. While these correlations do not demonstrate direct mechanistic interactions, they may reflect coordinated metabolic and cell cycle responses to infection-induced stress. The association between lysine degradation intermediates and the metalloreductase STEAP4 further connects this metabolic remodeling to iron-centered host defense mechanisms, highlighting the coordinated nature of the splenic immunometabolic response during *V. anguillarum* infection. Among the proposed candidate biomarkers, methylsuccinic acid may represent an early stage candidate biomarker for vibriosis. As an intermediate of propanoate metabolism, it may reflect early metabolic shifts preceding disease symptoms.^72^ Its correlation with cell-cycle regulators CDK1 and UHRF1 may reflect parallel metabolic and cell cycle responses to infection-associated stress. Negative correlations between piscibactin and host proteins involved in lysosomal proteolysis and RNA splicing (e.g., TPP1 and SNU13) point to broader stress responses associated with infection and iron imbalance, potentially reflecting host–pathogen interaction interfaces. Similar alterations in lysosomal function, proteostasis, and RNA processing have been reported during infection- and iron-associated cellular stress responses.^11,12,73^ These findings should be interpreted with caution, as the observed associations and AUC values derived from the same sample cohort require further experimental validation in independent *in vivo* cohorts.

The combined detection of host iron-regulatory proteins (e.g., STEAP4), the pathogen-derived siderophore piscibactin, and infection-response metabolites (e.g., 2-aminoadipic acid, saccharopine, and methylsuccinic acid) supports the utility of integrated multiomics approaches for identifying infection-associated molecular signatures. Integrating host- and pathogen-derived features may improve specificity over single-marker approaches. Beyond their well-established role in virulence, siderophores also offer diagnostic potential, with profiling enabling highly specific, noninvasive pathogen identification, even in mixed infections.^74^ Given its conservation among fish- and mollusk-pathogenic bacteria,^23,46^ piscibactin represents a promising candidate for future validation as an infection-associated marker.

In conclusion, this study provides a systems-level characterization of the splenic response of *S. senegalensis* to *V. anguillarum* infection. By integrating metabolomic and proteomic data sets, we revealed coordinated alterations associated with iron homeostasis, immunometabolic remodeling, and pathogen-derived siderophore production during vibriosis. These findings provide new insight into the molecular basis of disease in this important aquaculture species. Future validation studies incorporating longitudinal sampling and natural infection models will further refine our understanding of host–pathogen interactions and support the development of molecular tools for disease monitoring in aquaculture.

## Supplementary Material







## Data Availability

Raw metabolomics data files have been deposited on the *MetaboLights* server under accession number *MTBLS11240*.^75^ The mass spectrometry proteomics data have been deposited to the ProteomeXchange Consortium via the PRIDE partner repository with the data set identifier *PXD068174*.^76^

## Notes

Additional details on data analysis and supplementary figures and tables (PDF)
Tables with annotated differentially expressed metabolites (XLS)
Tables with annotated differentially expressed proteins (XLSX)

## #

Institute for Biomedicine and Glycomics, Griffith University, Parklands Drive, Southport, Australia
