# Supplementary material for: Integrated Metabolomic and Proteomic Analyses Reveal Altered Iron Homeostasis and Immunometabolic Remodeling in the Spleen of Solea senegalensis during Vibriosis
Source: J Agric Food Chem. 2026 Oct 19;74(43):34468–80. doi: 10.1021/acs.jafc.6c02961 (PMC13741842; doi:10.1021/acs.jafc.6c02961)
Supplement: Supplementary file 1 [file jf6c02961_si_001.pdf]

Supporting information

Integrated Metabolomic and Proteomic Analyses Reveal Altered Iron Homeostasis and Immunometabolic Remodeling in the Spleen of *Solea senegalensis* During Vibriosis

Larissa Buedenbender,[1,4] Miguel Balado,[2,5] Diego Rey-Varela,[2] Marta A. Lages,[2] Patricia Fernández Puente,[3] Jaime Rodríguez,[1*] Manuel L. Lemos,[2] Anthony R. Carroll,[4] and Carlos Jiménez[1*]*

[1] Departmento de Química, Facultad de Ciencias and CICA - Centro Interdisciplinar de Química e Bioloxía, Universidade da Coruña, A Coruña 15071, Spain.

[2] Department of Microbiology and Parasitology, Aquatic One Health Research Center (ARCUS), University of Santiago de Compostela, Santiago de Compostela 15782, Spain.

[3] Proteomics Laboratory, CICA - Centro Interdisciplinar de Química e Bioloxía, Universidade da Coruña, A Coruña 15071, Spain.

[4] School of Environment and Science, Griffith University, Southport, QLD, 4222, Australia.

[5] Department of Aquaculture and Blue Biotechnology, Centro Oceanográfico de Vigo (COV), Instituto Español de Oceanografía - Consejo Superior de Investigaciones Científicas (IEO-CSIC), Vigo 36390, Spain.

* Corresponding Authors:

Carlos Jiménez (carlos.jimenez@udc.es), Jaime Rodríguez (jaime.rodriguez@udc.es) and Miguel Balado (miguel.balado@ieo.csic.es)

Table of content

Figure S1. TSA-1 plates streaked with spleens of *S. senegalensis* (A) control group - injected with PSB buffer, (B) Low infection group - injected with 10^4 cells/fish of *V. anguillarum* (C) High infection group - injected with 10^5 cells/fish of *V. anguillarum* (D) *S. senegalensis* exhibiting external signs of vibriosis. 2

Figure S2. (A) sPLS-DA classification error rates of positive ionization mode metabolomics data (B) sPLS-DA classification error rates of negative ionization mode metabolomics data. 3

Figure S3. (A) Volcano plot showing the DEMs in the low infection group vs the control group in positive ionisation mode. (B) Volcano plot showing the DEMs in the high infection group vs the control group in positive ionisation mode. (C) Volcano plot showing the DEMs in the low infection group vs the control group in negative ionisation mode. (D) Volcano plot showing the DEMs in the high infection group vs the control group in negative ionisation mode. All volcano plots were based on |fold change| > 2.0, adjusted *p*-value <0.05. 3

Figure S4. Overview of annotated metabolite classes of the significant DEMs....4

Figure S5. GO enrichment for category Biological Process....4

Figure S6. GO enrichment for category Cellular Component. 5

Figure S7. GO enrichment for category Molecular Function. 5

Figure S8. Diagnostic plot from multiblock sPLS-DA....6

Figure S9. Classification error rates (overall and balanced) to identify the number of components. 6

Figure S10. Loading variables of components 1-3 identified as relevant for the separation between the different treatment groups (Control, Low and High) by the multi-block DIABLO model. The colour code refers to the treatment group, in which the respective variable has a higher median value....7

Figure S11. Correlation network visualising relevant connections (cut-off > 0.85) between significant variables in component 1-3 of the proteomics and metabolomics data blocks. The node colour and shape represent the different data blocks: mustard rectangles - proteomics; purple ellipses - metabolomics. Pink lines indicate that positive correlations and blue lines show negative correlations. 8

Figure S12. ROC curves of important metabolites identified via DIABLO analysis in *S. senegalensis* infected with *V. anguillarum.* Box plots and ROC analyses of metabolites with the highest biomarker potential are shown in the main text. 8

Figure S13. ROC curves of important proteins identified via DIABLO analysis in *S. senegalensis* infected with *V. anguillarum.* Box plots and ROC analyses of proteins with the highest biomarker potential are shown in the main text. 9

Table S1. MS-DIAL (v. 4.9.221218) data pre-processing parameters 10

Statistical methods 12

Multi-Omics Integration with MixOmics R package 12

References 12

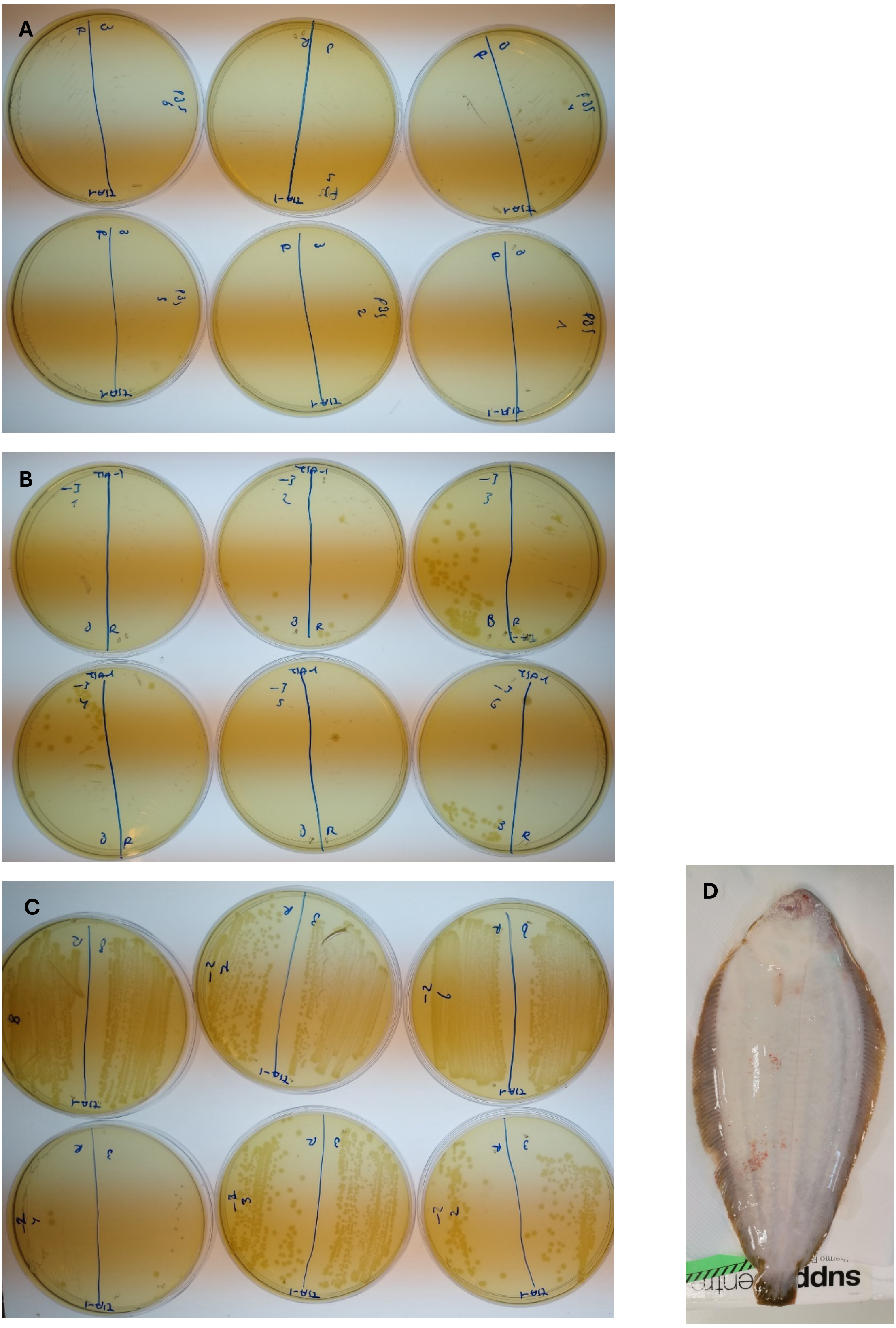

Figure S1. TSA-1 plates streaked with spleens of *S. senegalensis* (A) control group - injected with PSB buffer, (B) Low infection group - injected with 10^4 cells/fish of *V. anguillarum* (C) High infection group - injected with 10^5 cells/fish of *V. anguillarum* (D) *S. senegalensis* exhibiting external signs of vibriosis.

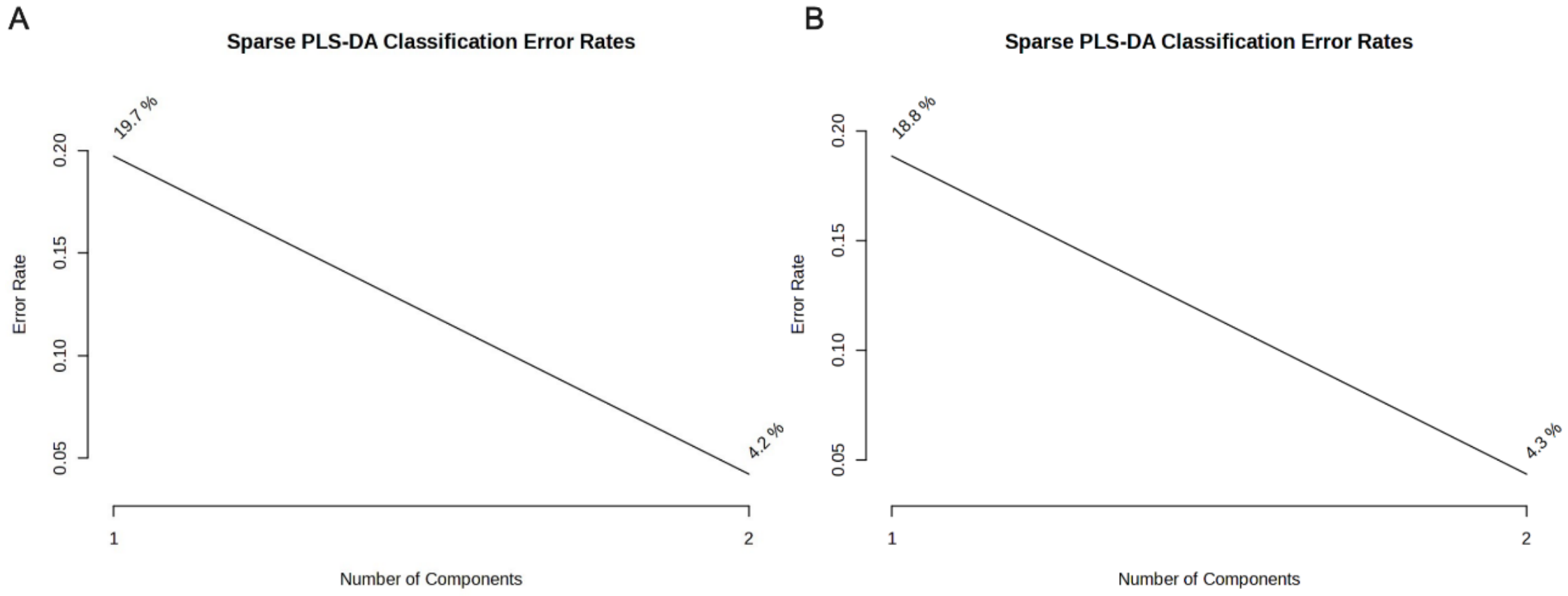

Figure S2. (A) sPLS-DA classification error rates of positive ionization mode metabolomics data (B) sPLS-DA classification error rates of negative ionization mode metabolomics data.

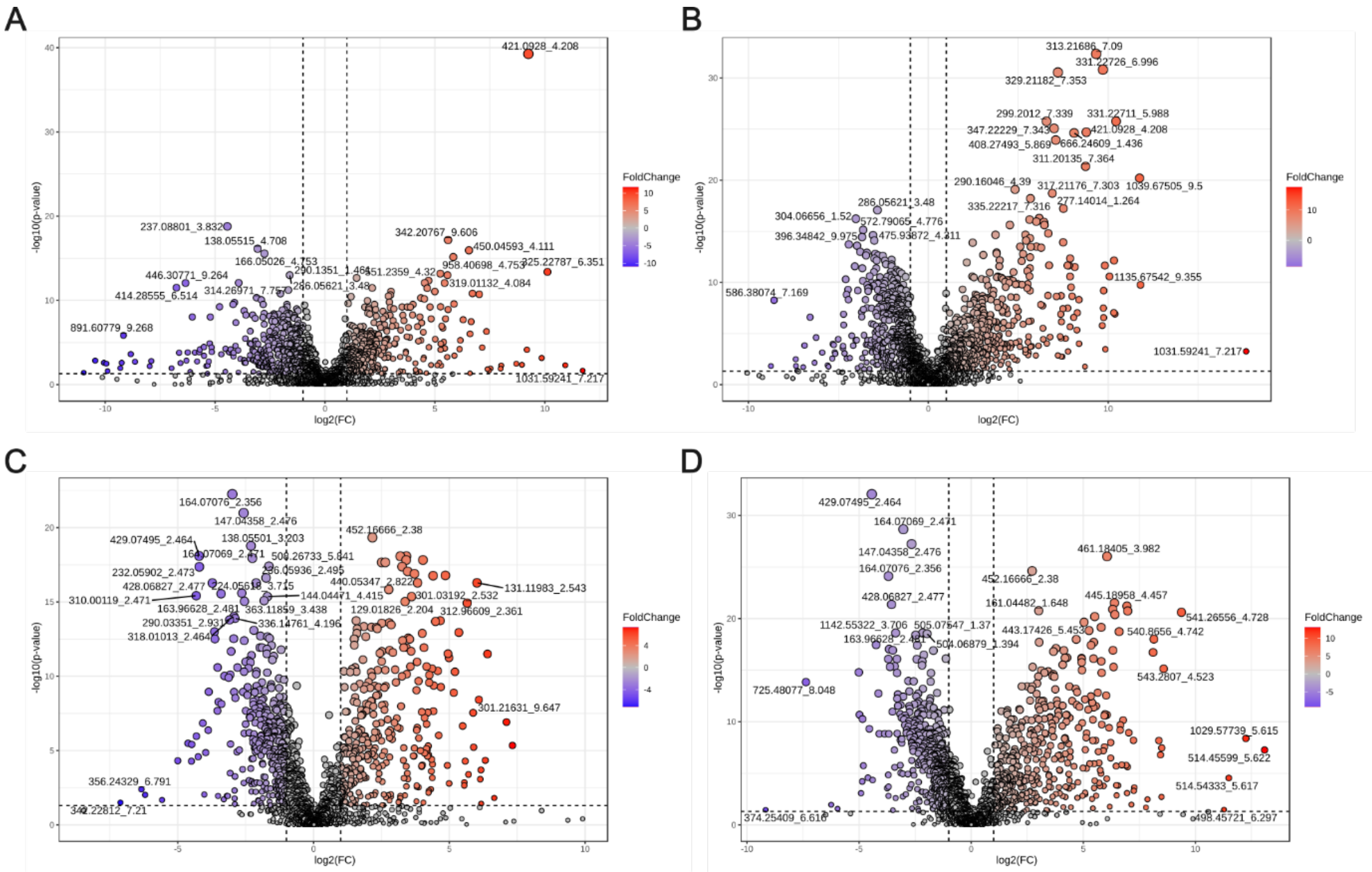

Figure S3. (A) Volcano plot showing the DEMs in the low infection group vs the control group in positive ionisation mode. (B) Volcano plot showing the DEMs in the high infection group vs the control group in positive ionisation mode. (C) Volcano plot showing the DEMs in the low infection group vs the control group in negative ionisation mode. (D) Volcano plot showing the DEMs in the high infection group vs the control group in negative ionisation mode. All volcano plots were based on |fold change| > 2.0, adjusted p-value <0.05.

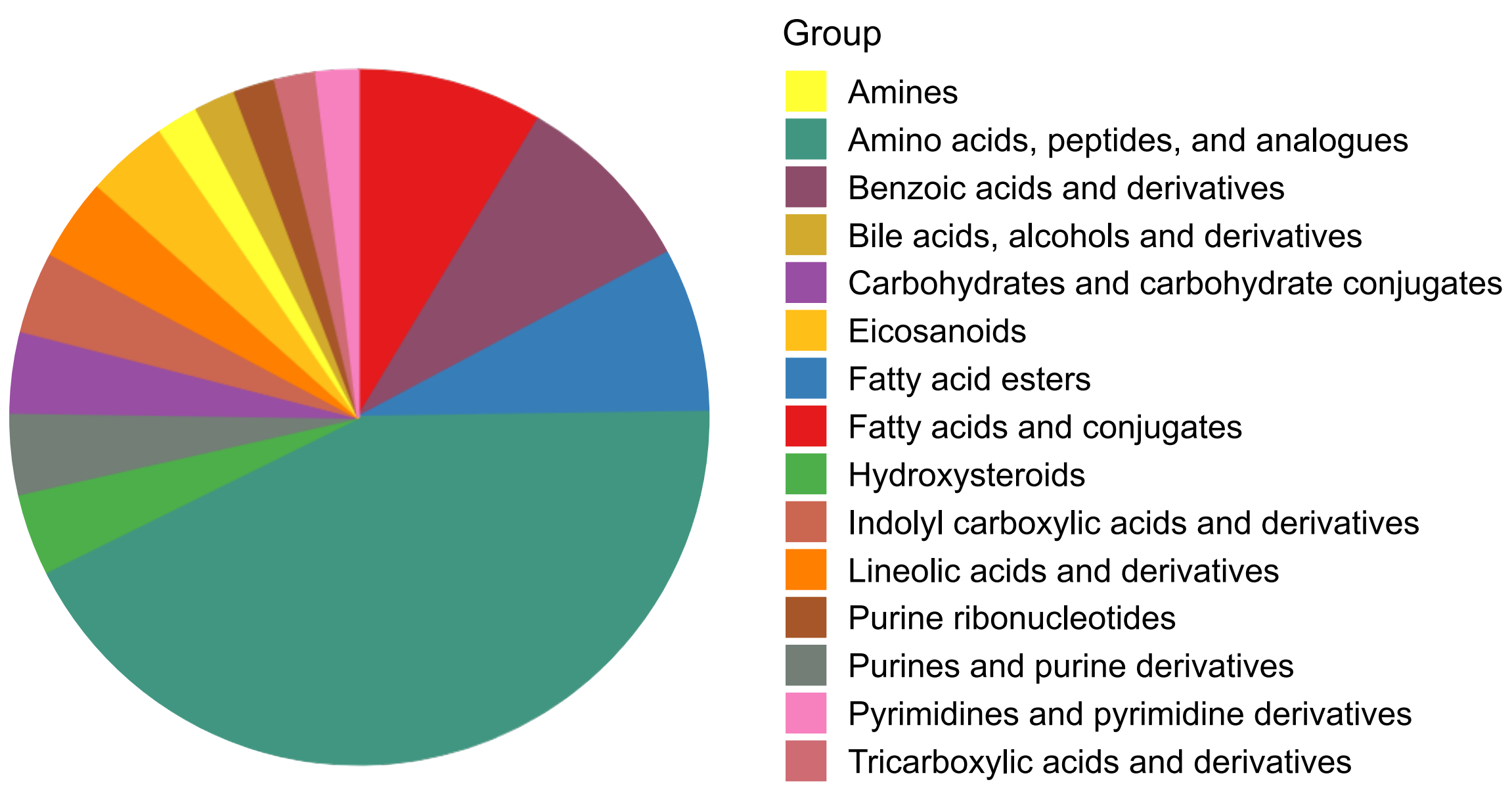

Figure S4. Overview of annotated metabolite classes of the significant DEMs.

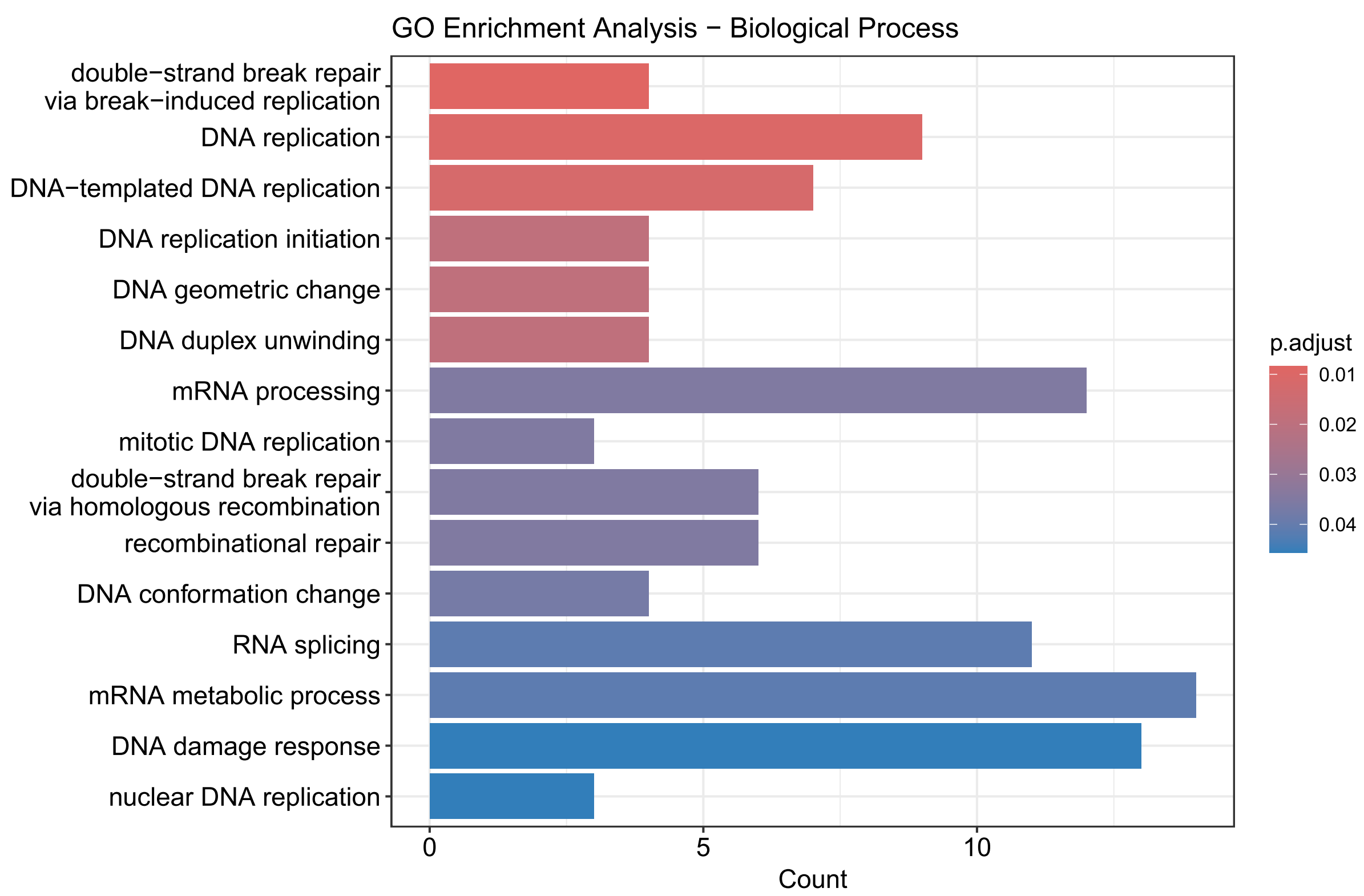

Figure S5. GO enrichment for category Biological Process.

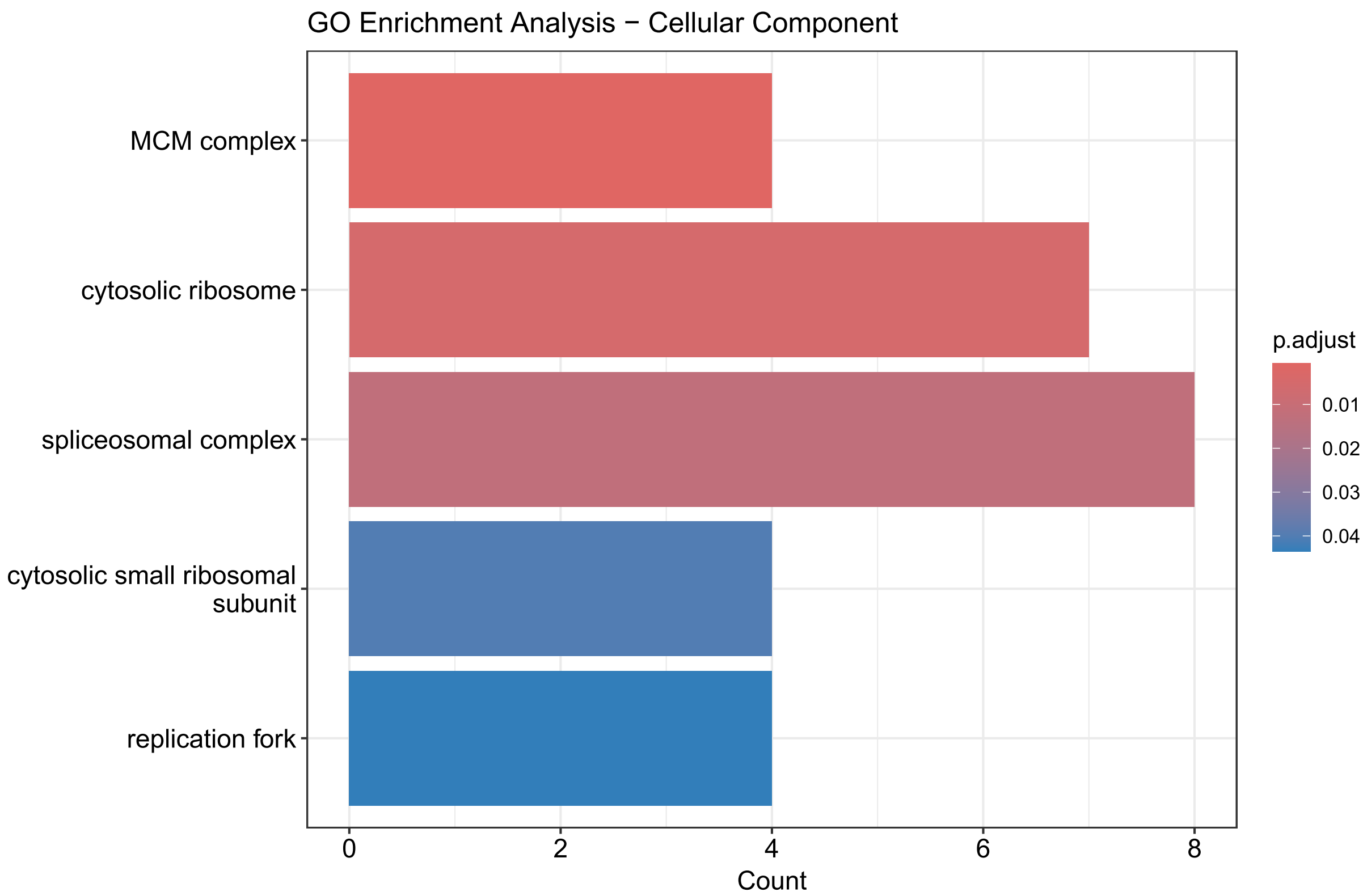

Figure S6. GO enrichment for category Cellular Component.

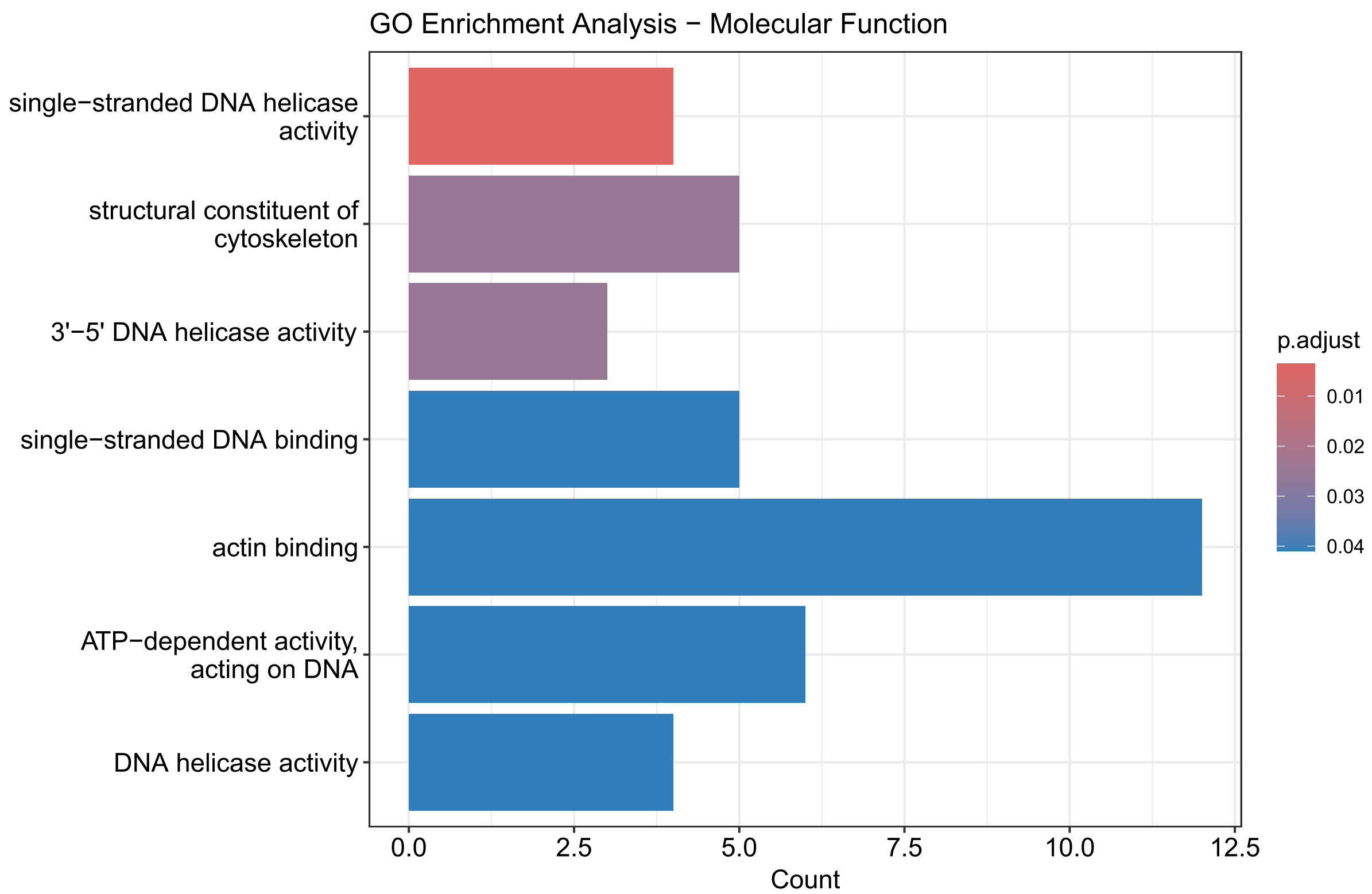

Figure S7. GO enrichment for category Molecular Function.

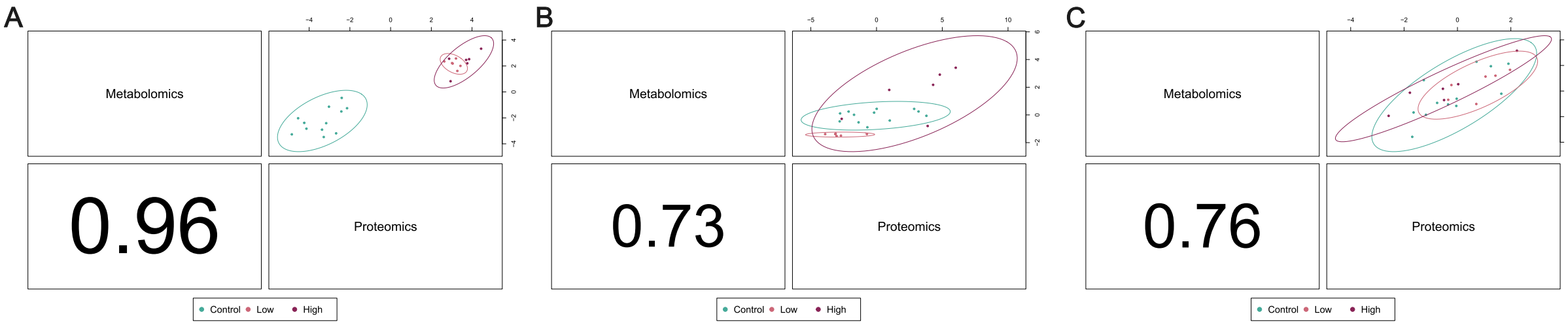

Figure S8. Diagnostic plot from multiblock sPLS-DA.

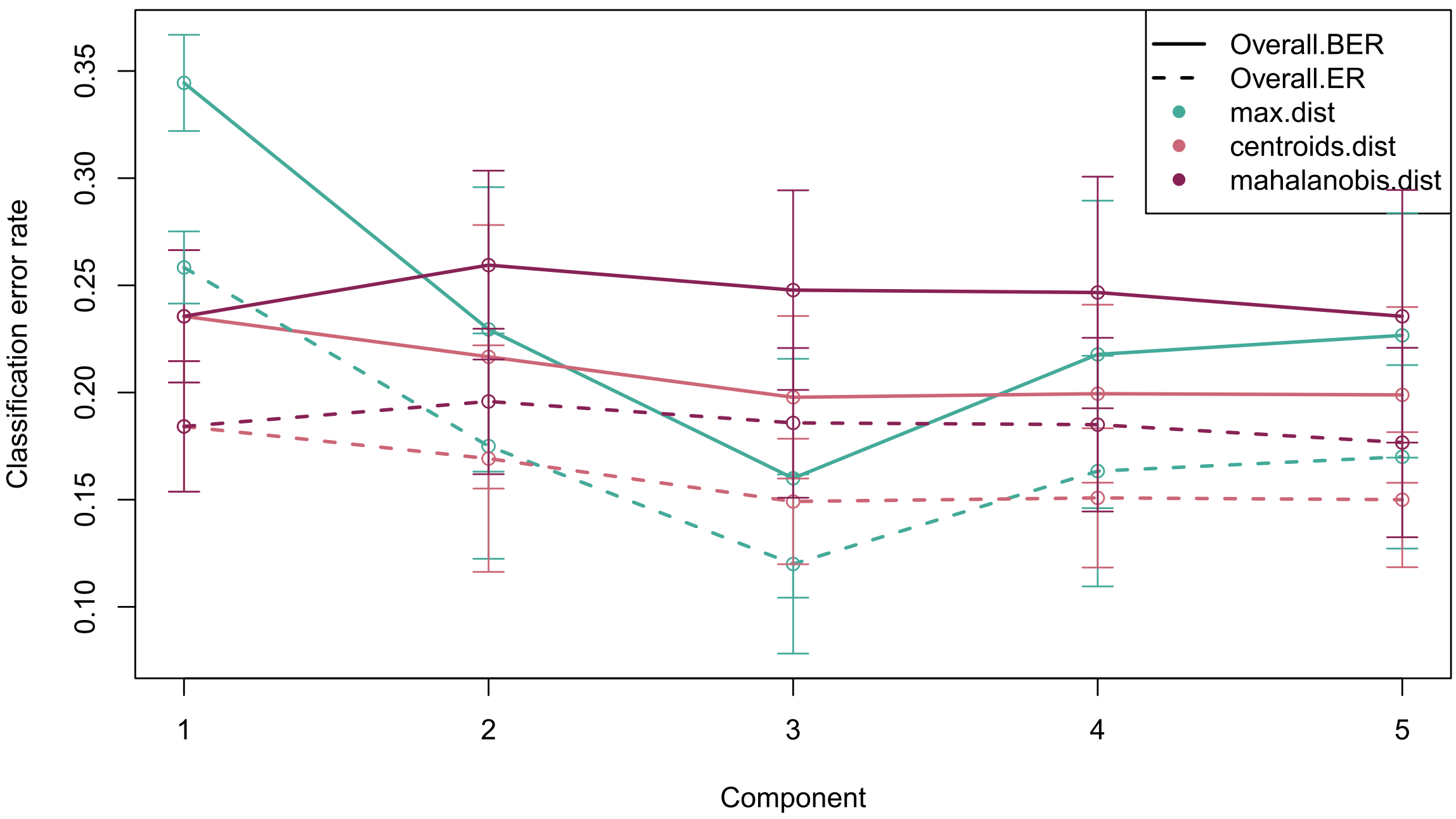

Figure S9. Classification error rates (overall and balanced) to identify the number of components.

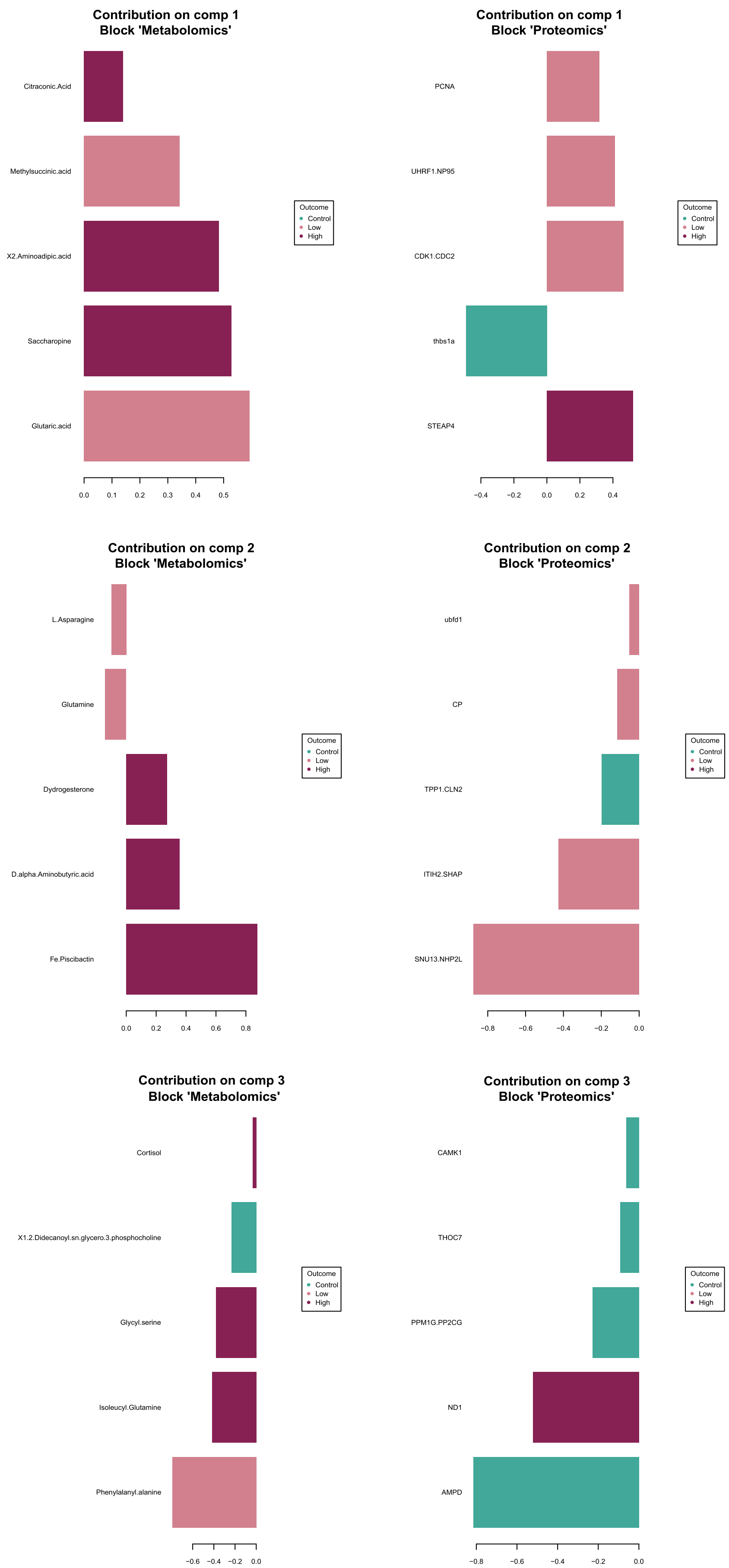

Figure S10. Loading variables of components 1-3 identified as relevant for the separation between the different treatment groups (Control, Low and High) by the multi-block DIABLO model. The colour code refers to the treatment group, in which the respective variable has a higher median value.

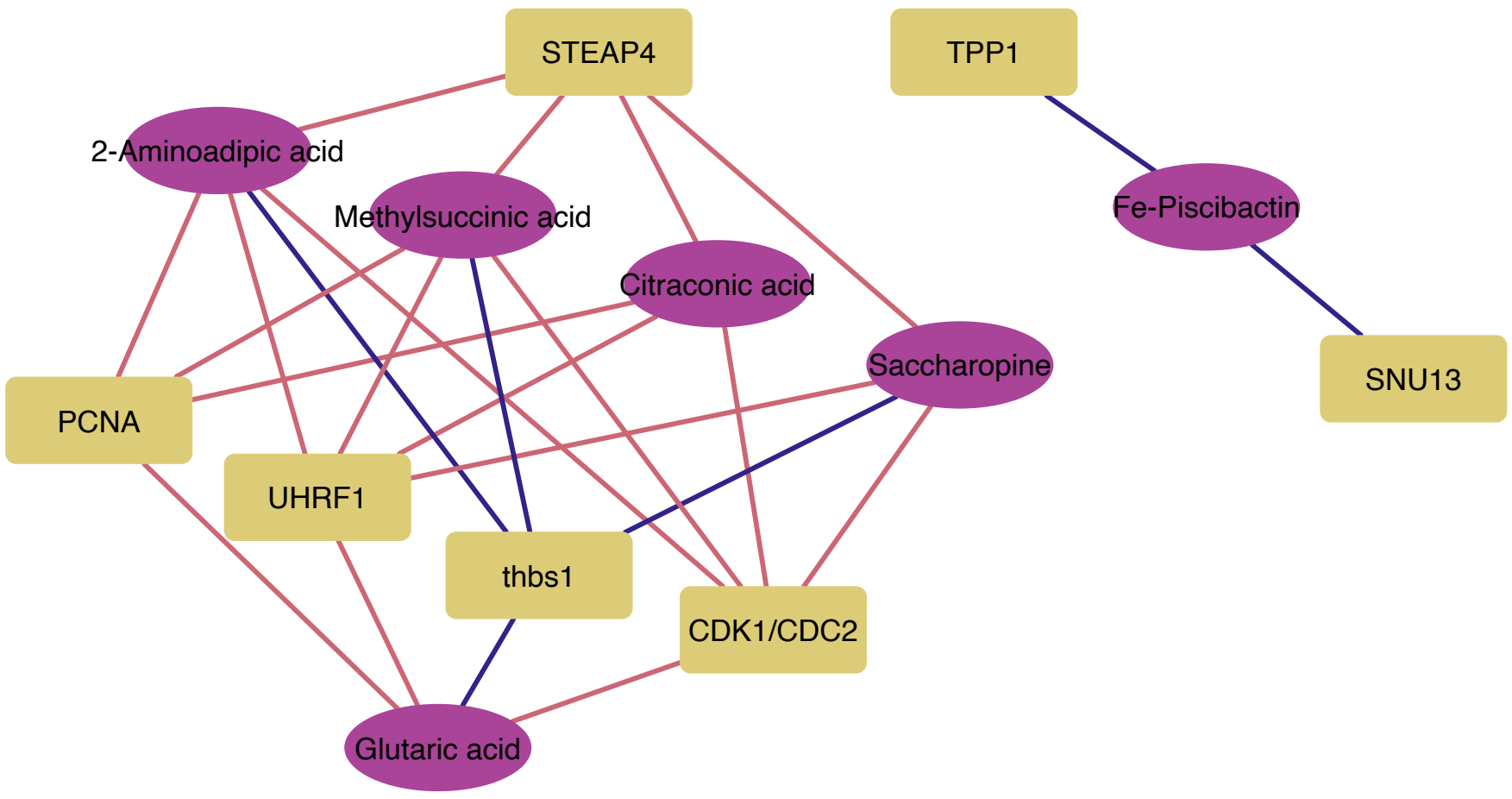

Figure S11. Correlation network visualising relevant connections (cut-off > 0.85) between significant variables in component 1-3 of the proteomics and metabolomics data blocks. The node colour and shape represent the different data blocks: mustard rectangles - proteomics; purple ellipses - metabolomics. Pink lines indicate that positive correlations and blue lines show negative correlations.

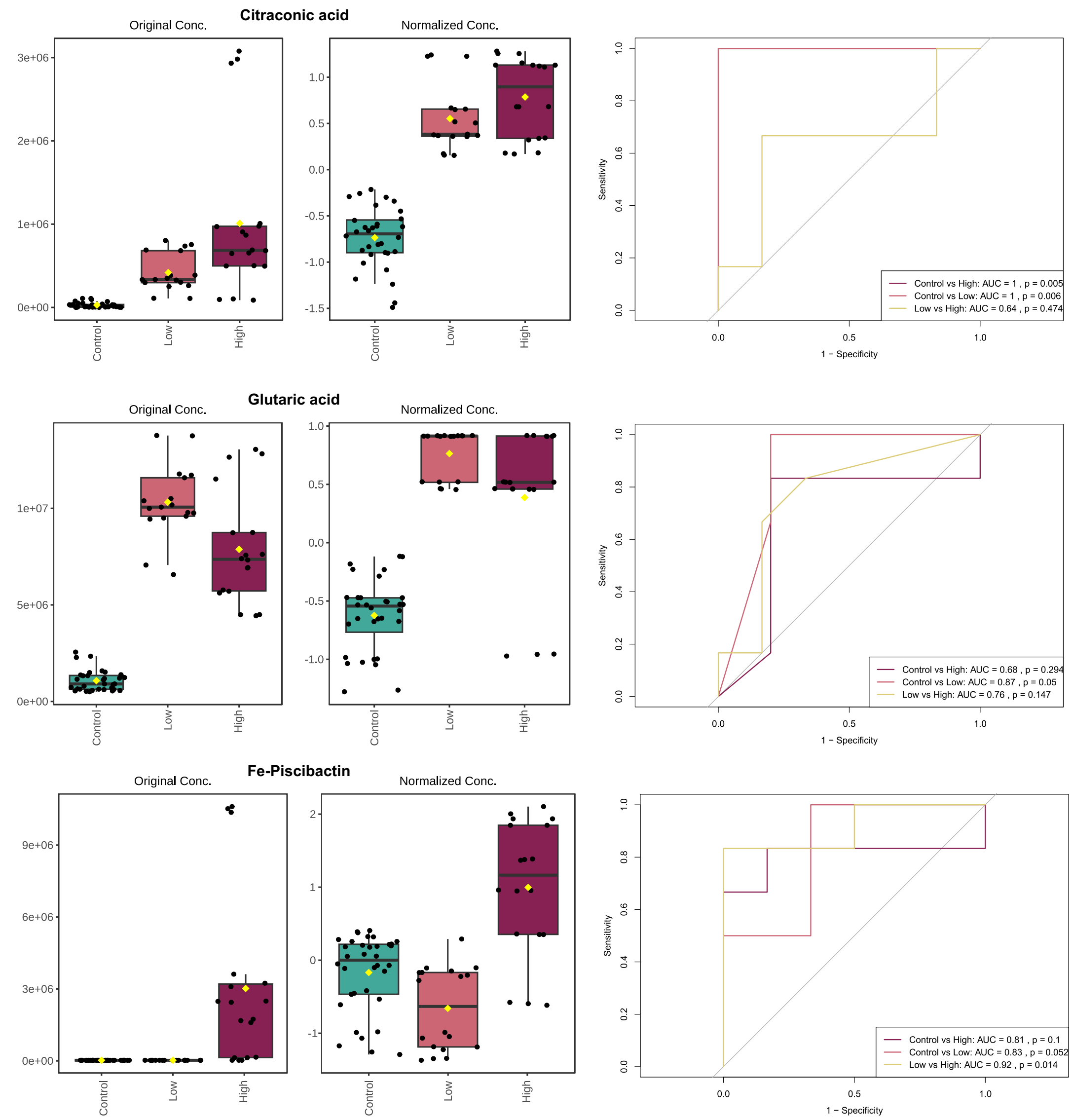

Figure S12. ROC curves of important metabolites identified via DIABLO analysis in *S. senegalensis* infected with *V. anguillarum.* Box plots and ROC analyses of metabolites with the highest biomarker potential are shown in the main text.

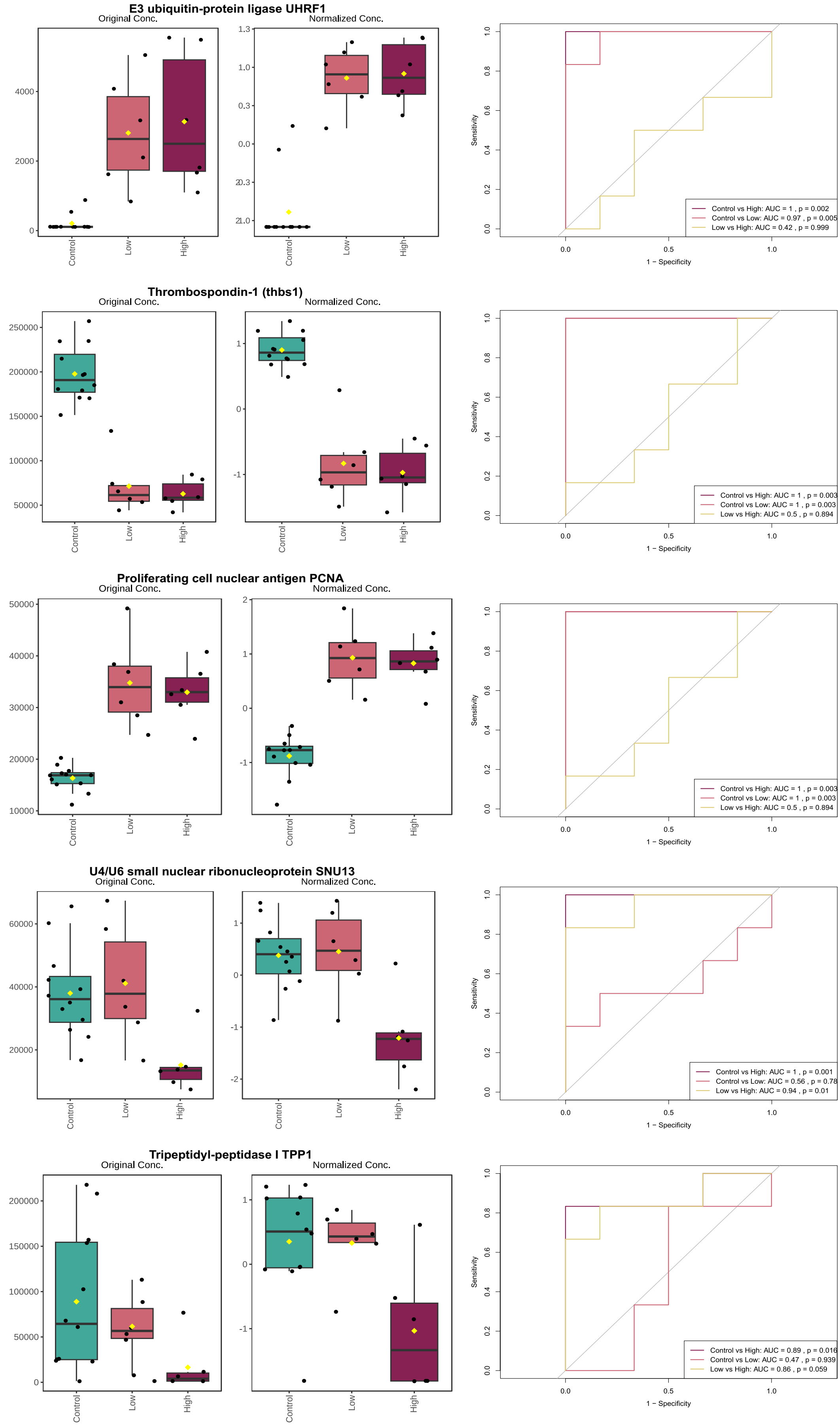

Figure S13. ROC curves of important proteins identified via DIABLO analysis in *S. senegalensis* infected with *V. anguillarum*. Box plots and ROC analyses of proteins with the highest biomarker potential are shown in the main text.

Table S1. MS-DIAL (v. 4.9.221218) data pre-processing parameters

#Project
MS1 Data type Centroid
MS2 Data type Centroid
Ion mode Positive
Target Metabolomics
Mode diMSMS

#Data collection parameters
Retention time begin 0.5
Retention time end 12.5
Mass range begin 80
Mass range end 1200
MS2 mass range begin 0
MS2 mass range end 1200

#Centroid parameters
MS1 tolerance 0.01
MS2 tolerance 0.05

#Isotope recognition
Maximum charged number 2

#Data processing
Number of threads 4

#Peak detection parameters
Smoothing method LinearWeightedMovingAverage
Smoothing level 3
Minimum peak width 5
Minimum peak height 3000

#Peak spotting parameters
Mass slice width 0.1
Exclusion mass list (mass & tolerance)

#Deconvolution parameters
Sigma window value 0.6
MS2Dec amplitude cut off 100
Exclude after precursor True
Keep isotope until 0.5
Keep original precursor isotopes False

#MSP file and MS/MS identification setting
MSP file MoNA-export-LC-MS-MS_Positive_Mode.msp
Retention time tolerance 100
Accurate mass tolerance (MS1) 0.01
Accurate mass tolerance (MS2) 0.05
Identification score cut off 70
Using retention time for scoring False
Using retention time for filtering False

#Adduct ion setting
$[M+H]^+$
$[M+Na]^+$

$[M+K]^+$
$[M+ACN+H]^+$
$[M+H-H_2O]^+$
$[2M+H]^+$
$[2M+Na]^+$
$[2M+K]^+$

#Alignment parameters setting
Reference file QC_2AFAMM.abf
Retention time tolerance 0.5
MS1 tolerance 0.015
Retention time factor 0.5
MS1 factor 0.7
Peak count filter 0
N% detected in at least one group 25
Remove feature based on peak height fold-change True
Sample max / blank average 5
Sample average / blank average 5
Keep identified and annotated metabolites False
Keep removable features and assign the tag for checking False
Gap filling by compulsion True

#Tracking of isotope labels
Tracking of isotopic labels FALSE

#Ion mobility
Ion mobility data FALSE

Statistical methods

The metabolomics datasets both for positive and negative ionisation mode were quartile normalised, log-transformed and pareto-scaled. Sample SL4_M2_R1 (1st replicate) in positive mode and samples SC0_M6_R3 and SL4_M2_R3 in negative mode were removed from the dataset due to missed injection (low sample volume in MS vial).

Multi-Omics Integration with MixOmics R package

In a multi-omics approach, the significant annotated metabolites from the untargeted metabolomics data were correlated with the significant proteins derived from the shotgun proteomics data. The two omics-datasets were considered as blocks in the multi-omics analysis, containing n = 191 entries in the metabolomics block, and n = 225 entries in the proteomics block. Data Integration Analysis for Biomarker discovery using Latent cOmponents (DIABLO) for omics studies was used for combining the metabolomic and proteomic datasets [1]. The DIABLO algorithm relies on the identification of a limited number of correlated variables from multiple datasets to predict their influence on an outcome (in our study infection by *V. anguillarum*) [1]. We defined a design matrix to specify the structure of the data integration in the DIABLO analysis. Therefore, we first assessed the correlation between the metabolites and proteins via a sPLS model which showed a high degree of correlation with a value of 0.847. This correlation coefficient was used as the reference value for generating the data matrix on which the DIABLO analysis was based. Cross-validation was conducted using 6-folds to address class representation issues, and the performance of the model was evaluated using 50 repeats to ensure reliability (Figure S9). The CV results indicated a lower error rate with inclusion of three components for a more reliable and generalizable model. Hence, the DIABLO model was assessed for 3 components and 5 variables in each component. The correlation coefficient (r = 0.96) for component 1 showed a very strong correlation across the metabolomics and proteomics datasets and explained a significant portion of the variance and relationship between the infected and control groups (Figure S8). The results were presented in a Circos plot that was built on a similarity matrix and represents the correlation between variables from different data blocks. The cut-off was set to 0.85 to only show the most significant correlations between proteins and metabolites.

References

[1] F. Rohart, B. Gautier, A. Singh, K.A. Lê Cao, mixOmics: An R package for ‘omics feature selection and multiple data integration, *PLoS Comput Biol* **2017**, *13* (11), e1005752. https://doi.org/10.1371/journal.pcbi.1005752.
